# Cross-cultural evidence for positive control and mind-mindedness as buffers for parental distress

**DOI:** 10.1038/s44271-026-00484-x

**Published:** 2026-06-17

**Authors:** Claire Hughes, Elian Fink, Ayalguu Agtchin, Laure Lu Chen, Caoimhe Dempsey, Rory T. Devine, Hana D’Souza, Miryam Edwards, Louise Gray, Jean Anne Heng, Mikeda Jess, Mishika Mehrotra, Siu Ching Wong, Catherine Wu, Jiayin Zheng, Zhen Wu, Zhenlin Wang, Chengyi Xu

**Affiliations:** 1 Centre for Child, Adolescent and Family Research, University of Cambridge, Cambridge, UK; 2 School of Psychology, University of Sussex, Brighton, UK; 3 Department of Psychological and Cognitive Sciences, Tsinghua University, Beijing, China; 4 Department of Applied Social Sciences, The Hong Kong Polytechnic University, Hong Kong, China; 5 School of Psychology, University of Birmingham, Birmingham, UK; 6 School of Psychology, Cardiff University, Cardiff, UK; 7 Faculty of Education, University of Cambridge, Cambridge, UK; 8 Department of Psychology, The Education University of Hong Kong, Hong Kong, China; 9 Institute of Education, Massey University, Auckland & Palmerston North, New Zealand

**Keywords:** Human behaviour, Risk factors

## Abstract

Parental distress impacts children’s emotional adjustment, highlighting the need to identify protective factors. Promising candidates include positive control (i.e., autonomy support, indexed by parental encouragement, praise, and open-ended questions) and representational mind-mindedness (the propensity for parents to view children as independent agents). To counterbalance the focus on European and North American samples, we included 849 parent-child dyads from England, Hong Kong and Mainland China (*M*_child age_ = 5.17 years, *SD* = 0.51). Parents rated their own distress and child internalizing problems; their interactions with their children were assessed for positive control, and their speech samples were coded for representational mind-mindedness. In a single-paper meta-analysis, parental distress was associated with elevated child internalizing problems and low positive control consistently across sites, but only associated with low mind-mindedness in England. Within-site analyses showed that intergenerational associations in distress-internalizing problems were attenuated by positive control in Mainland China and by mind-mindedness in England; multi-group modeling demonstrated that this latter effect was site-specific. These findings highlight the importance of widening the cultural scope of this research field.

## Abstract (web summary)

Across England, Hong Kong and Mainland China (*N* = 849) parental distress is linked to child internalizing problems. Yet protective factors differ across contexts, as this link is attenuated by positive control in China, but by mindmindedness in England.

## Introduction

Child internalizing problems (i.e., symptoms of anxiety, social withdrawal and low mood) often co-occur with parental distress^1^, predict negative long-term outcomes such as mood disorders^2^ and persist over time if left untreated^3^. The rising prevalence of internalizing problems^4,5^ makes identifying potential protective factors an important research challenge. Here, we consider two possible parental buffers that may mitigate the risk of a child internalizing problems: positive control and mind-mindedness. Positive control, the use of praise, questions and encouragement, is typically assessed via observations of guided learning^6^. In North American and European cultures, positive control is diametrically opposed to parental intrusiveness and associated with measures of both parental mental health and child adjustment^7^. Parental mind-mindedness, defined as the propensity to view children as agents with their own thoughts and feelings^8^, is known to attenuate associations between risk factors and child externalizing problems^9,10^ (see also Nieto-Retuerto et al.^11^ for a recent meta-analytic review of the wider literature on parental mentalizing and children’s externalizing problems). However, the mitigating effects of mind-mindedness regarding child internalizing problems remain poorly understood.

Globally, the lifetime prevalence of major depressive disorder varies from 2% to 21%: findings from different research designs indicate that East Asia and Europe show the lowest and highest reported rates, respectively^12,13^. However, as is typical for developmental science^14^, studies of the familial transmission of internalizing problems have focused heavily on North American and European cultures. Responding to a recent call for a ‘chronospatial revolution’^15^ to augment the scope, data, synergy and theoretical framing within psychological research, our study includes direct observations of parent-child interactions for participants from England, Mainland China and Hong Kong (which has its own distinct socio-political system^16^). Our study is framed by the view that parenting shows cultural universality without uniformity. That is, core aspects of parenting may be widely shared but vary in both function and form. In terms of function, the same behavior, such as vocalization, can promote either interdependence or independent exploration. Likewise, the form of parental behaviors can also show site differences (e.g., affection can be displayed physically via hugs/kisses or more indirectly, via educational support). This view builds on evidence for contrasting parenting approaches in East Asian and European, North American and Australian cultures^17^. Specifically, our three-site multi-method design enabled us to examine whether, across cultures, the emotional impact of parental distress on children is buffered in similar or distinct ways by positive control and mind-mindedness.

Alongside expanding the geographical scope of research on mind-mindedness, by focusing on children making the transition to school, our current study increases this field’s developmental scope, as previous studies have focused heavily on infancy, toddlerhood and the early preschool years^18^. Recruiting children at the transition to school also enabled us to investigate a period in which children face multiple new situations and social challenges that can elicit feelings of both anxiety and depression^19^. Meta-analytic findings on parental mentalizing and child externalizing problems^11^ supports the promise of this developmental focus, as links between parental mentalizing (indexed either by mind-mindedness or by the broader measure of parental reflective functioning) and child externalizing problems are stronger in middle childhood (*r* = -.33) than in early childhood (*r* = -.11) or adolescence (*r* = -.13). As these authors note, these age-related contrasts may reflect differences in both children’s developmental competencies and in the relative salience of contextual effects, as environmental support is likely to matter most during periods of transition, such as starting school.

This introductory section is structured around three questions. In the first and second parts, we ask whether, across cultures, parental distress is linked with (a) child internalizing problems, and (b) reduced positive control and mind-mindedness. Third, we ask whether positive control and parental mind-mindedness attenuate the association between parental distress and child internalizing problems.

### Across cultures, is parental distress related to child internalizing problems?

Alongside evidence for the intergenerational transmission of internalizing problems^20,21^, genetically sensitive studies highlight the importance of environmental pathways, including parental modeling, on child’s internalizing problems^22^. Child internalizing problems relate to multiple aspects of parenting, including over-involved and over-reactive parenting, and reduced authoritative parenting^23^. While adult anxiety and depression are often studied separately, there is growing support for an alternative dimensional approach that includes each of these indicators of parental distress^24^. Reflecting cultural influences on parenting^17^, the association between parental distress and child internalizing problems may vary across sites. However, existing evidence is very limited in geographical scope. For example, of the 19 studies included in Rose and colleagues’ systematic review of links between parenting and child internalizing problems, 17 involved samples from North America, Europe or Australia^23^.

Fortunately, recent multi-site investigations are expanding the cultural scope of this field. In a study of 173 families with young adolescents from the UK or Türkiye, associations between parental distress and child internalizing problems were similar across sites, leading the authors to suggest that ‘mindful parenting’ is ‘culture-free’^25^. Likewise, a survey-based study of 17-year-olds (*N* = 12,757) from ten cultures (i.e., China, Czech Republic, Hungary, the Netherlands, Slovenia, Spain, Switzerland, Taiwan, Turkey, and the United States) showed a culturally-universal link between parenting processes and adolescent mental health^26^. However, another 9-site survey-based study of 1315 families with young adolescents (i.e., China, Colombia, Italy, Jordan, Kenya, the Philippines, Sweden, Thailand, and the United States) showed that associations between parental rejection and child internalizing/externalizing problems were stronger in sites with higher normative levels of parental rejection^27^. Highlighting the relevance of this reported moderation effect for the current study, recent findings from a longitudinal study of 841 Pakistani mother-toddler dyads show that harsh parenting (closely related to rejection) mediates the link between maternal depression and children’s socio-emotional adjustment^28^. Together, these findings support the universality–without-uniformity perspective^29^, in which common patterns in parenting are expected across sites, but with specific processes showing differing relative salience.

A useful framework for understanding why associations between parental distress and child internalizing problems might be culturally invariant is self-determination theory^30^, which posits universal links between well-being and the satisfaction of three basic psychological needs (for autonomy, competence and relatedness). Although not originally a developmental theory, self-determination theory has proved useful in parenting research^31^. Examples of empirical support for this perspective come from evidence that (a) prenatal need satisfaction predicts Belgian mothers’ postnatal wellbeing^32^ and (b) parental distress constrains parental support for these basic psychological needs. For example, anxious parents who engage in over-controlling behavior to reduce uncertainty may constrain child autonomy, a pattern reflected in research on links between ‘helicopter parenting’ and youth anxiety and depression^33^. Likewise, if anxious parents model avoidance behaviors, children may build a sense of the world as threatening, depleting their sense of themselves as competent^34^. And parents who feel overwhelmed may struggle to respond to their child, reducing children’s sense of connectedness. Together, these pathways underscore potentially universal benefits of positive control (i.e., autonomy support) and mind-mindedness (i.e., attunement to the child’s inner states) for limiting the emotional impact of exposure to parental distress.

### Is parental distress related to reduced positive control and mind-mindedness?

Meta-analytic reviews suggest that well-being underpins effective parenting for both mothers^7^ and fathers^35^. However, the underlying mechanisms have proved complex and difficult to elucidate. As noted in a review of 152 studies^36^, these mechanisms encompass multiple pathways that are *motivational* (e.g., reduced child-oriented goals), *cognitive* (e.g., limited attention to child input), and *emotional* (e.g., reduced positive affect). This multi-path model supports a differentiated model of parenting in which protection, control and guided learning represent three key dimensions^37^, each of which is likely to contribute to parental use of positive control. Longitudinal evidence from American^38^ and British^39^ samples confirms that parental distress constrains positive parenting.

Theoretically, distress is also expected to compromise mind-mindedness, because the self-focus associated with depression^40^ may constrain parents’ ability to tune into their child’s mental states. Empirical support for this view comes from studies involving mothers with infants^41,42^ and preschoolers^43^. However, other studies report moderating effects for both child and contextual characteristics. For example, in a longitudinal study of 50 Turkish mothers, prenatal depression showed no overall association with interactive mind-mindedness at 12-month post-partum, but there was a significant interaction effect, such that prenatal depression was only associated with reduced mind-mindedness in the context of low infant negative emotionality^44^. Likewise, in a study involving 65 Italian mothers of both full-term and premature infants, parenting stress showed no main effect association with interactive mind-mindedness at 6-months postpartum, but was associated with reduced mind-mindedness if mothers received medium or high levels of social support^45^. That is, maternal perception of emotional support from close others promoted mind-mindedness, but only in the context of low or mild parenting stress. Together, these findings suggest that tuning into others’ thoughts and feelings may be difficult under conditions of stress, even in the absence of symptoms of depression or anxiety.

### Do positive control and parental mind-mindedness attenuate the association between parental distress and child internalizing problems?

According to Grolnick and Pomerantz^46^, capturing the complexity of parental socialization requires going beyond the traditional view of control as authoritarianism to adopt a multiple-forms approach that encompasses positive aspects of control that can facilitate children’s competence. One example is structuring, which has been reported to underpin moderating effects of neighborhood risk on the impact of parental restrictions^47^. Structuring is also known as ‘behavioral control’^29^, which overlaps with the construct of ‘positive control’ (i.e., using praise and encouragement to guide child activities and maintain task focus). Expanding this prior work on within-culture contextual contrasts, we will compare whether the mitigating effects of positive control vary across study sites.

For example, a study of parenting across 13 cultural groups in Africa, Asia, Europe, the Middle East, and North and South America showed that parental control is associated with hostility in European American families, but with care and concern within East Asian cultures^48^. Yet culture is dynamic in nature, and recent evidence suggests cross-cultural similarities in parental influences on child internalizing problems^26^. However, the widespread reliance on single-informant ratings constrains the strength of this conclusion. To address this issue of non-independence, in the current study, we observed parent-child interactions using the *Etch-a-Sketch Online*^49^, an adaptation of a widely-used cooperation paradigm^50^. As parental involvement in child activities is a primary expression of care in Mainland China, we anticipated that positive control would play an especially salient role in reducing the risk for internalizing problems in this site.

Turning to mind-mindedness, cultural accounts of mind-behavior orientation suggest that East Asian contexts, deeply influenced by philosophical traditions such as Confucianism, tend to emphasize social harmony, conformity, and behavioral regulation, and may place relatively less emphasis on explicitly referencing young children’s internal mental states while prioritizing observable behavior^51^. Supporting this view, separate studies have reported that, compared with parents in England or Australia, parents in Japan, Hong Kong and Mainland China display lower average levels of representational and interactive mind-mindedness^52–54^. Yet, these contrasts are accompanied by between-site similarities in the correlates of mind-mindedness^54^. This combination of between-site contrasts in mean levels and within-site similarities in correlates may reflect the fact that within-culture differences in parenting are often larger than between-culture differences^55^. As such, mind-mindedness may play a similar function across different sites, despite marked site contrasts in mean levels^56^. Supporting this view, our previous findings demonstrate that, across sites, within-site individual differences in mind-mindedness show similarly sized associations with individual differences in (a) positive control^6^ and (b) prenatal bonding and safety from violence^57^.

Parental autonomy support requires an active consideration of the child’s perspective (i.e., mind-mindedness), but work is still needed to integrate mind-mindedness and self-determination theory. Drawing on the model that wellbeing hinges on the satisfaction of basic psychological needs for relatedness, autonomy and competence, we posit that child internalizing problems are likely to be reduced when parents who, despite their own problems of depression or anxiety, can tune into their child’s interests and feelings. A proclivity to focus on mental states will also shape how parents perceive and respond to children’s behaviors, reducing the propensity to label child behaviors as irritating or difficult^58^. These pathways may help explain how parental mind-mindedness might reduce associations between risk factors and externalizing problems^9,10^. Here, we hypothesize that mind-mindedness will also attenuate the impact of familial risk for child internalizing problems.

### The current study

In sum, the current multi-method study included a large sample of parent-child dyads from three sites (England, Hong Kong and Mainland China). Our first two research questions concerned whether parental psychological distress (hereafter, parental distress) was associated, in a culturally-universal pattern across sites, with (a) child internalizing problems and (b) parental positive control and mind-mindedness. Building on evidence that *co-occurring* symptoms of depression and anxiety are associated with reduced sensitivity and positive control in guided learning^59^, we expected to find a significant inverse association between observational ratings of parental positive control and our multi-measure index of parental anxiety, stress and depressive symptoms. Conversely, as noted earlier, associations between parental distress and mind-mindedness appear attenuated in the context of stress. As our data collection took place during a time of global stress related to the Covid-19 pandemic (2021–2022), we expected parental distress to show only weak inverse associations with mind-mindedness. Our third question concerned whether, in each site, positive control and/or mind-mindedness attenuated the expected intergenerational link between parental distress and child internalizing problems. Our decision to conduct parallel analyses in each site was guided by the fact that, while we were able to demonstrate across-site measurement invariance for our survey-based measures of parental distress and child internalizing problems, this was not possible for our observational ratings of these two potential protective factors.

## Methods

### Participants

As part of a larger study of children’s well-being and socio-cognitive development, 849 parent-child dyads were recruited via social media and schools in England (ENG, *n* = 259), Hong Kong (HK, *n* = 266), and Mainland China (MC, *n* = 324) (see Table 1 for demographic details; note that parent and child gender were based on how each participant identified). The English sample was primarily White (70%), with 9% identifying as mixed/multiple ethnic groups, 5% as Asian British, 1% as Black British, and 15% undisclosed. The Hong Kong sample and the Mainland Chinese sample were predominantly Han Chinese ( > 90%). To account for site differences in child age, *F*(2, 838) = 41.35, *p* < 0.001, parent age, *F*(2, 839) = 62.20, *p* < 0.001, parent degree-level education, *F*(2, 844) = 65.55, *p* < 0.001, and perceived social standing, *F*(2, 829) = 11.68, *p* < .001, these child and parent characteristics were included as covariates in our analyses.

**Table 1:** Participant demographic information

	ENG (*n* = 259)	HK (*n* = 266)	MC (*n* = 324)	Effect sizes for group differences
*Child*				
Age (*M*, SD)	5.26 (0.37)	4.94 (0.36)	5.28 (0.64)	*η*^2^ = 0.090 [0.055, 0.127]
Range	4.42–6.52 years	4.00–5.74 years	4.01–6.49 years	
Gender (% female)	127 (49)	129 (48)	155 (48)	Cramér’s *V* = 0.010 [0.000, 0.034]
Gender (% male)	132 (51)	137 (52)	169 (52)	
*Parent*				
Age (*M*, SD)	38.44 (4.53)	37.88 (3.98)	34.92 (3.93)	*η*^2^ = 0.129 [0.089, 0.170]
Range	20–54 years	28–53 years	23–47 years	
Gender (% female)	243 (94)	238 (90)	308 (95)	Cramér’s *V* = 0.082 [0.000, 0.144]
Gender (% male)	16 (6)	26 (10)	16 (5)	
Parent education (% degree-level)	223 (86)	147 (55)	269 (83)	*η*^2^ = 0.134 [0.094, 0.176]
Perceived social standing (*M*, SD)	6.53 (1.40)	6.23 (1.53)	5.94 (1.48)	*η*^2^ = 0.027 [0.009, 0.051]

Parent education (% degree-level) = percentage of parents having a bachelor’s degree or higher. Perceived social standing = parents’ perceived standing on a 10-rung social ladder. All child and parent participants identified as either male or female; 95% confidence intervals are presented in brackets.

*ENG* England, *HK* Hong Kong, *MC* Mainland China.

### Procedure

With approval from the Research Ethics Committees of the University of Cambridge, the University of Birmingham, the Education University of Hong Kong, and Tsinghua University, data collection took place between June 2021 and December 2022. In each site, we obtained written informed consent from parents and verbal assent from children. Trained researchers followed a detailed manual to ensure identical procedures across sites. To comply with Covid-19-related social restrictions, our protocol included remote home-based observations (via *Zoom* in England and Hong Kong, and *Tencent Meeting* in Mainland China). Five bilingual researchers translated the study measures into Cantonese and Mandarin Chinese, following a collaborative iterative process to ensure conceptual equivalence.

### Measures

#### Parental positive control

Parents were observed interacting with their child using an online observation paradigm, *Etch-a-Sketch Online*^49^. Mirroring the traditional Etch-a-Sketch task where two dials control either horizontal or vertical lines, the online version requires the child to press the ‘A’ (left) or ‘D’ (right) keys to draw horizontally, and the parent to press the ‘O’ (up) or ‘M’ (down) keys to draw vertically. Dyads were given 5 min to copy a target image (an envelope or a house) displayed onscreen, and video footage was coded using the Parent-Child Interaction System (PARCHISY)^60^. In this challenging task, parental positive control (e.g., use of praise, explanations, and open-ended questions) was rated on a 7-point scale (1 = no occurrence, 7 = continual occurrenc*e*). Native speakers, including at least one senior coder, double-coded each video. Across sites, inter-rater reliability was high (ICCs > 0.80).

#### Parental representational mind-mindedness

Parents described their child and their relationship with the child via the Five-Minute Speech Sample paradigm^61^. Parents were given 5 min to describe their child and how they get along. They were instructed: “*I’d like to hear your thoughts and feelings about [CHILD], in your own words. Please speak for five minutes, telling me what kind of a person [CHILD] is and how the two of you get along*.” If parents stopped speaking before the five-minute point, they were given a standardized prompt (“*You have another X minutes. Can you say anything else about [CHILD]?*”). Originally developed for clinical studies, the Five-Minute Speech Sample paradigm is now widely used in family research as a simple and effective method for capturing parents’ thoughts and feelings about their child^62^. Transcripts can be coded for a range of parental representations, including narrative coherence^63^, family affective attitudes^64^, and aspects of parental mentalizing such as reflective functioning^65^ and mind-mindedness^54^.

In this study, in each site, trained native-speaking researchers transcribed and coded anonymized audio-recordings for mind-mindedness^66^. Child-focused attributes were coded as either mental (e.g., “she is a joyful child” or “he loves solving puzzles”) or non-mental (e.g., “she’s very tall” or “he’s 5 years old”). Exact repetitions were excluded. Across the three sites, inter-rater reliability, based on 20% of the speech samples, was good for both mental (ICCs > 0.88) and non-mental attributes (ICCs > 0.82). Meta-analytic evidence shows that child outcomes display modest links with both frequency counts and proportion scores of mind-mindedness, with somewhat stronger predictive utility for frequency counts^18^. Accordingly, we used the frequency count of mental attributes in parents’ descriptions of their child to index mind-mindedness. Additionally, as in previous studies^54^, we accounted for parental verbosity by including the frequency of non-mental attributes as a covariate in subsequent analyses.

#### Parental distress

We administered three validated measures to capture diverse aspects of parents’ distress. Each item on the 10-item Kessler Psychological Distress Scale (K10) refers to the last 4 weeks and is rated on a 5-point scale (0 = none of the time, 4 = all of the time)^67^. Total scores range from 0 to 40, with higher scores reflecting greater levels of nonspecific psychological distress. The 9-item Patient Health Questionnaire (PHQ-9) refers to the last 2 weeks and includes somatic disturbance and low mood^68^. The 7-item Generalized Anxiety Disorder Scale (GAD-7) also refers to the last 2 weeks and is focused on worries, anxiety, and irritability^69^. For both the PHQ-9 and the GAD-7, items are rated on a 4-point scale (0 = not at all, 3 = nearly every day), yielding total scores ranging from 0 to 27 for the PHQ-9 and from 0 to 21 for the GAD-7. We established partial scalar invariance for each of the three measures across sites (see Supplementary Materials); Table S1 summarizes the internal consistency (Cronbach’s *α*s) and the full results of the measurement invariance analyses.

#### Child internalizing problems

Parents reported on child internalizing problems using the 5-item Emotional Symptoms (e.g., “many worries, often seems worried”) and 5-item Peer Problems (e.g., “rather solitary, tends to play alone”) subscales of the Strengths and Difficulties Questionnaire (SDQ)^70^. In the SDQ framework, internalizing problems are conventionally operationalized as the combination of these two subscales^71^. The SDQ demonstrates acceptable internal consistency across culturally diverse parent samples (Cronbach’s *α* = 0.66)^72^. Parents rate how true each item is for their child on a 3-point scale (0 = not true, 1 = somewhat true, 2 = very true). Internal consistencies in the English and Hong Kong samples were acceptable, Cronbach’s *α*s = 0.65–0.70. Consistent with prior research using the Chinese version of the SDQ (Cronbach’s *α* = 0.57)^73^, Cronbach’s *α* value was 0.54 in the Mainland Chinese sample. Based on our previous work establishing partial scalar invariance of the SDQ across these three sites^74^, we dropped four items (good friend, popularity, best with adults, somatic symptoms) from the internalizing problems dimension. This exclusion increased internal consistency in the Mainland Chinese sample to 0.61. Table S2 provides a detailed summary of the SDQ items selected to model the internalizing latent factor. Table S3 summarizes the full results of measurement invariance analyses. Total scores range from 0 to 12, with higher scores indicating greater levels of internalizing problems.

#### Control variables

We included both child and parent characteristics (age and gender), as well as family socioeconomic status (SES), as covariates. To index family SES, parents reported their highest level of educational attainment (1 *= *junior secondary school or below, 2 = senior secondary school, 3 = associate degree, 4 = bachelor’s degree, 5 = master’s degree, 6 = PhD) and completed the Ladder of Subjective Social Status^75^ by rating their perceived social standing within their local cultural context on a 10-rung ladder, where the top represents those with the highest levels of educational qualification, income status, and employment and the bottom represents those with the lowest.

### Analysis plan

The study aims and analyses presented here were pre-reregistered in May 2025 at https://osf.io/kfxuc/registrations. Descriptive analyses and data screening were conducted using *SPSS* Version 29, while all subsequent main analyses were performed in *R* and M*plus* Version 8^76^. To address our first two research questions, we conducted a single-paper meta-analysis^77^ to examine whether the association between parental distress and child internalizing problems and mind-mindedness/positive control was consistent across sites. Partial correlations (pr) were used as standardized, covariate-adjusted effect sizes to enable tests of heterogeneity across sites. This analysis used a random-effects model via the *robumeta*^78^, *metafor*^79^, and *dplyr*^80^ packages in *R*. To address our third research question, moderation analyses and simple slope tests were carried out using a robust maximum likelihood estimator in M*plus*, which accounts for non-normal distributions^81^. No participants were excluded from the dataset. Assumptions of the statistical models were examined prior to analysis, and no substantial violations were detected (Figs. S1 and S2). We applied a full information approach to retain all available cases in the analyses^82^. Unlike listwise deletion or mean imputation, this approach does not assume multivariate normality or completely random missingness, and thus yields less biased parameter estimates and standard errors^82^.

## Results

### Descriptive statistics and data reduction

Table 2 presents descriptive statistics for the main study measures. Although not the primary focus of the present study, significant between-site differences were observed in nonspecific psychological distress as indexed by the K10 (HK > MC ≈ ENG), in both parental positive control and mind-mindedness (ENG > HK > MC), and in child internalizing problems (ENG ≈ HK > MC). To simplify the main analyses, we used confirmatory factor analyses to derive factor scores for parental distress and child internalizing problems^83^. First, we specified a just-identified measurement model in which three observed indicators (i.e., the total scores of the K10, PHQ-9, and GAD-7) loaded significantly onto a single latent factor representing parental distress (for standardized estimates, see Table S4). Using scale totals as indicators allowed us to integrate the three measures while maintaining a parsimonious measurement model. Second, as reported elsewhere^74^ (see also Table S3), we specified a measurement model in which each of the six items loaded significantly onto a single latent factor representing internalizing problems (for standardized estimates, see Table S5). This model provided a good fit to the data from England, *χ*^2^(8) = 15.85, *p* = 0.045, RMSEA = 0.06, CFI = 0.95, TLI = 0.91, Hong Kong, *χ*^2^(8) = 15.32, *p* = 0.053, RMSEA = 0.06, CFI = 0.95, TLI = 0.91, and Mainland China, *χ*^2^(8) = 6.06, *p* = 0.640, RMSEA = 0.00, CFI = 1.00, TLI = 1.00. The factor determinacy coefficients were ≥ 0.95 for parental distress and ≥ 0.81 for internalizing problems, indicating high internal consistency^81^.

**Table 2:** Descriptive statistics

	ENG			HK			MC			
	*M* (SD)	Range	*n*	*M* (S*D*)	Range	*n*	*M* (SD)	Rang*e*	*n*	*F* ^*p*^
Mind-mindedness^a^	13.99 (7.62)	0–41	259	11.47 (6.34)	0–33	266	8.19 (4.62)	0–22	324	64.16 ^<0.001^
Positive control	4.53 (1.32)	1–7	225	4.03 (1.27)	2–7	242	2.35 (1.12)	1–6	324	244.71 ^<0.001^
Psychological distress^b^										
K10	7.79 (6.12)	0–35	253	9.85 (6.57)	0–31	260	8.66 (7.20)	0–40	324	6.12 ^0.^^002^
PHQ-9	4.51 (4.65)	0–27	249	3.82 (3.33)	0–20	260	3.98 (4.10)	0–27	324	2.02 ^0.^^134^
GAD-7	3.36 (3.59)	0–21	250	3.35 (3.49)	0–15	257	3.28 (3.74)	0–21	324	0.05 ^0.^^953^
Internalizing problems^c^	2.07 (1.99)	0–10	259	2.03 (1.84)	0–9	266	1.68 (1.74)	0–7	324	3.94 ^0.^^020^

*ENG* England, *HK* Hong Kong, *MC* Mainland China.

^a^Frequency of mental attributes.

^b^Total scores from each of the three questionnaire measures.

^c^Total scores on the 6-item internalizing subscale of the SDQ.

### Associations between parent distress and child internalizing problems

**Fig. 1:**
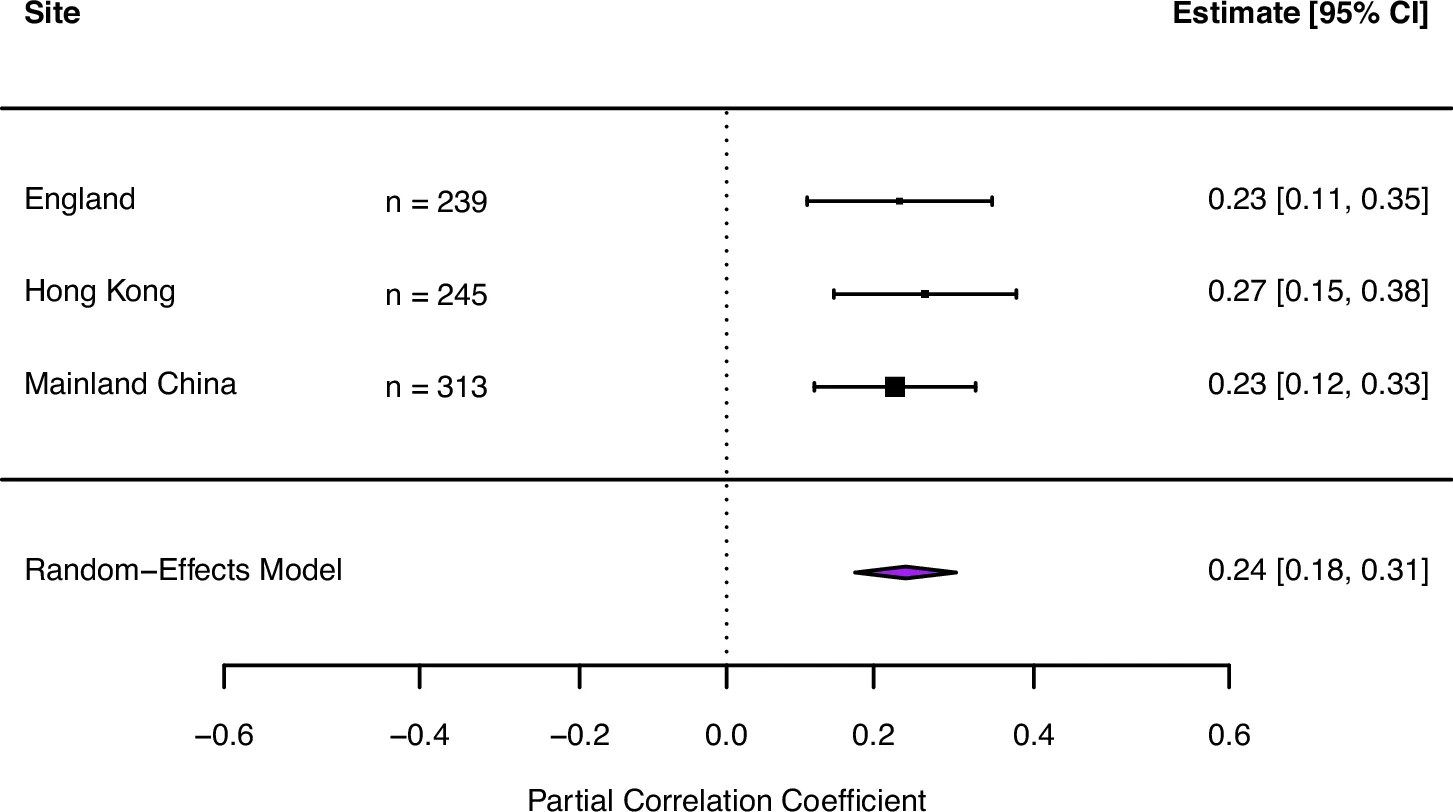
Forest plot displaying consistent effect sizes for the link between parental distress and child internalizing problems across sites. Bars represent 95% confidence intervals; square size reflects relative sample size; the diamond represents the overall effect estimated using a random-effects model.

Table 3 presents zero-order correlations among the key study variables, with corresponding confidence intervals reported in Table S6. Across allthree sites, parental distress showed a significant positive association with child internalizing problems; these associations held after controlling for child- and parent-level demographic covariates: England, pr(239) = 0.23, 95% CI [0.110, 0.350], *p* < 0.001; Hong Kong, pr(254) = 0.27, 95% CI [0.147, 0.380], *p* < 0.001; and Mainland China, pr(313) = 0.23, 95% CI [0.120, 0.331], *p* < 0.001. To assess the across-site consistency of this association, we conducted a single-paper meta-analysis, which offers key advantages for both the graphical representation and quantitative synthesis of within-study results^77^. As illustrated in Fig. 1, the partial correlation coefficients linking parental distress to child internalizing problems were highly consistent across sites, *Q*(2) = 0.26, *p* = 0.879. The single-paper meta-analysis revealed a statistically significant and robust positive association across sites, even when taking into account demographic covariates, with a summary estimate of pr = 0.24, 95% CI [0.177, 0.317], *p* < 0.001.

**Table 3:** Bivariate correlations

	1	2	3	4	5	6	7	8	9	10
*A. England*										
1. Mind-mindedness^a^	–									
2. Positive control	0.25**	–								
3. Psychological distress^b^	−0.16*	−0.19**	–							
4. Internalizing problems^c^	0.00	−0.09	0.25**	–						
*Covariates*										
5. Child age	0.11^+^	0.04	0.07	0.16*	–					
6. Child gender	−0.06	0.01	−0.02	0.07	0.11^+^	–				
7. Parent age	0.02	0.08	−0.06	−0.02	0.03	−0.08	–			
8. Parent gender	0.01	−0.01	−0.03	0.08	0.04	−0.01	−0.11^+^	–		
9. Parent education	0.11^+^	0.02	−0.10	0.05	0.00	−0.02	0.23**	−0.08	–	
10. Perceived social standing	0.08	0.02	−0.25**	−0.08	−0.04	0.04	0.21**	−0.09	0.35**	–
11. Non-mental frequency	0.40**	0.07	−0.11^+^	−0.09	−0.10	−0.01	0.05	0.08	0.11^+^	.03
*B. Hong Kong*										
1. Mind-mindedness^a^	–									
2. Positive control	0.18**	–								
3. Psychological distress^b^	0.11^+^	−0.02	–							
4. Internalizing problems^c^	0.03	0.05	0.25**	–						
*Covariates*										
5. Child age	0.03	−0.20**	−0.03	−0.15*	–					
6. Child gender	-0.09	-0.07	0.05	0.14*	0.06	–				
7. Parent age	0.19**	0.08	0.08	0.00	0.02	−0.06	–			
8. Parent gender	0.16**	0.03	0.10^+^	−0.08	0.00	0.04	−0.07	–		
9. Parent education	0.19**	0.28**	−0.10	−0.01	−0.08	−0.09	0.03	−0.09	–	
10. Perceived social standing	0.03	0.08	−0.34**	−0.01	−0.09	0.00	−0.11^+^	0.09	0.31**	–
11. Non-mental frequency	0.27**	0.00	−0.08	−0.06	0.02	−0.03	0.02	0.07	0.09	.05
*C. Mainland China*										
1. Mind-mindedness^a^	–									
2. Positive control	0.10^+^	–								
3. Psychological distress^b^	−0.08	−0.16**	–							
4. Internalizing problems^c^	0.04	−0.12*	0.26**	–						
*Covariates*										
5. Child age	0.09	−0.08	−0.21**	−0.09	–					
6. Child gender	0.07	0.10^+^	0.04	−0.05	0.00	–				
7. Parent age	0.04	0.11^+^	−0.08	−0.11*	0.14*	0.03	–			
8. Parent gender	−0.02	0.00	−0.02	−0.07	−0.02	−0.01	−0.04	–		
9. Parent education	0.16**	0.22**	−0.18**	−0.13*	−0.01	0.09	0.18**	−0.08	–	
10. Perceived social standing	0.10^+^	0.10^+^	−0.15**	−0.08	0.02	−0.07	−0.03	−0.04	0.25**	–
11. Non-mental frequency	0.59**	0.05	−0.04	0.06	−0.08	−0.09	0.06	0.05	0.05	0.03

Child and parent gender were dummy coded with male being the reference category.

**p < 0.01. *p < 0.05. ^+^*p* < 0.10.

^a^Frequency of mental attributes.

^b^Latent factor scores for psychological distress.

^c^Latent factor scores for internalizing problems.

### Associations between parental distress, mind-mindedness and positive control

The residual diagnostics supported the appropriateness of using linear models to estimate the associations between parental distress and mind-mindedness and between parental distress and positive control (see Figs. S1 and S2). As shown in Table 3, parental distress was unrelated to positive control in Hong Kong, pr(225) = −0.02, 95% CI [−0.154, 0.107], *p* = 0.715, but was inversely associated with positive control in England and Mainland China, even after controlling for demographic covariates: England, pr(211) = −0.20, 95% CI [−0.323, −0.063], *p* = 0.004; Mainland China, pr(313) = −0.15, 95% CI [−0.254, −0.037], *p* = 0.009. However, the single-paper meta-analysis indicated no significant differences in the strength of these partial correlations across sites, *Q*(2) = 3.56, *p* = 0.169, with a pooled estimate of −0.12, 95% CI [−0.220, −0.028], *p* = 0.011 (see Fig. S3).

Parental distress was inversely associated with mind-mindedness in England. After accounting for parents’ overall verbosity in speech samples (non-mental frequency counts) and demographic covariates (child age and gender; parent age, gender, education, and perceived social standing), this association remained significant but modest, pr(238) = −0.13, 95% CI [−0.250, 0.000], *p* = 0.049. In contrast, the corresponding associations were not significant in Mainland China or Hong Kong; a single-paper meta-analysis indicated that the strength of these associations differed significantly across the three sites, *Q*(2) = 7.39, *p* = 0.025, *I*² = 74% (see Fig. S4).

### Does mind-mindedness or positive control mitigate the intergenerational links between parental distress and child internalizing problems?

Across-site measurement invariance cannot be assessed for the observational measures of positive control (a 0–7 global rating) and representational mind-mindedness (a frequency count), because they are each single manifest indices. As the marked differences in mean scores also preclude the establishment of the utility of applying a common metric, we ran site-specific linear regressions and interpreted within-site effects. In each site, we tested whether positive control and mind-mindedness moderated the association between parental psychological distress and child internalizing problems. Collinearity between the two moderators was negligible, as variance inflation factors were approximately 1 across sites. Each model included mind-mindedness, positive control, psychological distress, and grand-mean centered interaction terms (mind-mindedness × distress; positive control × distress), controlling for all covariates^84^. All models were just identified. Table 4 presents the key model results (for baseline main-effect models, see Table S7). As a robustness check, Bayesian sensitivity analyses did not change the results (see Table S8).

**Table 4:** Robust maximum-likelihood estimates for the moderation models predicting children’s internalizing problems

	ENG				HK				MC			
	Est.	SE	Std.Est.	95% CI	Est.	SE	Std.Est.	95% CI	Est.	SE	Std.Est.	95% CI
Child age	0.100	0.089	0.080	[−0.059, 0.219]	**−0.102**	0.047	−0.143*	[−0.264, −0.021]	−0.015	0.018	−0.041	[−0.139, 0.056]
Child gender	0.086	0.055	0.099	[−0.026, 0.223]	**0.081**	0.034	0.158*	[0.030, 0.285]	−0.023	0.024	−0.051	[−0.152, 0.051]
Parent age	−0.001	0.006	−0.009	[−0.132, 0.115]	−0.001	0.004	−0.018	[−0.137, 0.101]	−0.004	0.004	−0.068	[−0.192, 0.056]
Parent gender	0.136	0.099	0.070	[−0.031, 0.170]	−0.110	0.061	−0.132	[−0.276, 0.011]	−0.080	0.063	−0.077	[−0.196, 0.041]
Parent education	0.039	0.029	0.082	[−0.038, 0.201]	−0.009	0.013	−0.041	[−0.159, 0.078]	−0.023	0.016	−0.081	[-.189, .027]
Perceived social standing	−0.017	0.023	−0.055	[−0.199, 0.090]	0.007	0.011	0.042	[−0.088, 0.171]	−0.006	0.010	−0.040	[−0.164, 0.083]
Non-mental frequency	−0.004	0.003	−0.096	[−0.233, 0.041]	−0.001	0.002	−0.019	[−0.145, 0.108]	0.001	0.003	0.016	[−0.116, 0.148]
Psychological distress	0.006	0.007	0.064	[−0.094, 0.222]	**0.012**	0.004	0.256**	[0.111, 0.402]	**0.007**	0.002	0.206**	[0.093, 0.318]
Mind-mindedness	0.001	0.005	0.025	[−0.150, 0.200]	0.003	0.004	0.081	[−0.089, 0.251]	0.004	0.003	0.089	[−0.030, 0.208]
Positive control	−0.022	0.021	−0.066	[−0.189, 0.056]	0.007	0.013	0.034	[−0.090, 0.157]	−0.016	0.011	−0.076	[−0.179, 0.026]
Mind-mindedness × Psychological distress	**−0.003**	0.001	−0.264**	[−0.449, −0.080]	0.000	0.001	−0.047	[−0.217, 0.123]	0.000	0.000	0.064	[−0.033, 0.161]
Positive control × Psychological distress	−0.004	0.004	−0.074	[−0.204, 0.055]	−0.004	0.003	−0.113	[−0.257, 0.031]	**−0.004**	0.002	−0.110*	[−0.212, −0.008]

Est. = unstandardized estimate; significant estimates are shown in bold. Std. Est. = standardized estimate; 95% CI = 95% confidence intervals for standardized estimates. Child and parent gender were dummy coded with male being the reference category.

*ENG* England, *HK* Hong Kong, *MC* Mainland China.

**p < 0.01; *p < 0.05.

In Mainland China (but not England or Hong Kong; see Table 4 for full statistics across all sites), there was a small but significant moderating effect of positive control on the link between parental distress and children’s internalizing problems, Std.Est. = −0.11, 95% CI = [−0.212, −0.008], *p* = 0.035. In England (but not Mainland China or Hong Kong; see Table 4 for full statistics across all sites), there was a small-to-moderate moderating effect of mind-mindedness on the link between parental distress and children’s internalizing problems, Std.Est. = −0.26, 95% CI = [−0.449, −0.080], *p* = 0.005. Moreover, pairwise *Wald* tests in a multi-group model indicated that the mind-mindedness moderation in England differed from both Hong Kong, *χ*²(1) = 4.51, *p* = 0.034, and Mainland China, *χ*²(1) = 8.96, *p* = 0.003. In contrast, the positive control moderation did not differ by sites, joint *Wald χ*²(2) = 0.03, *p* = 0.983.

**Fig. 2:**
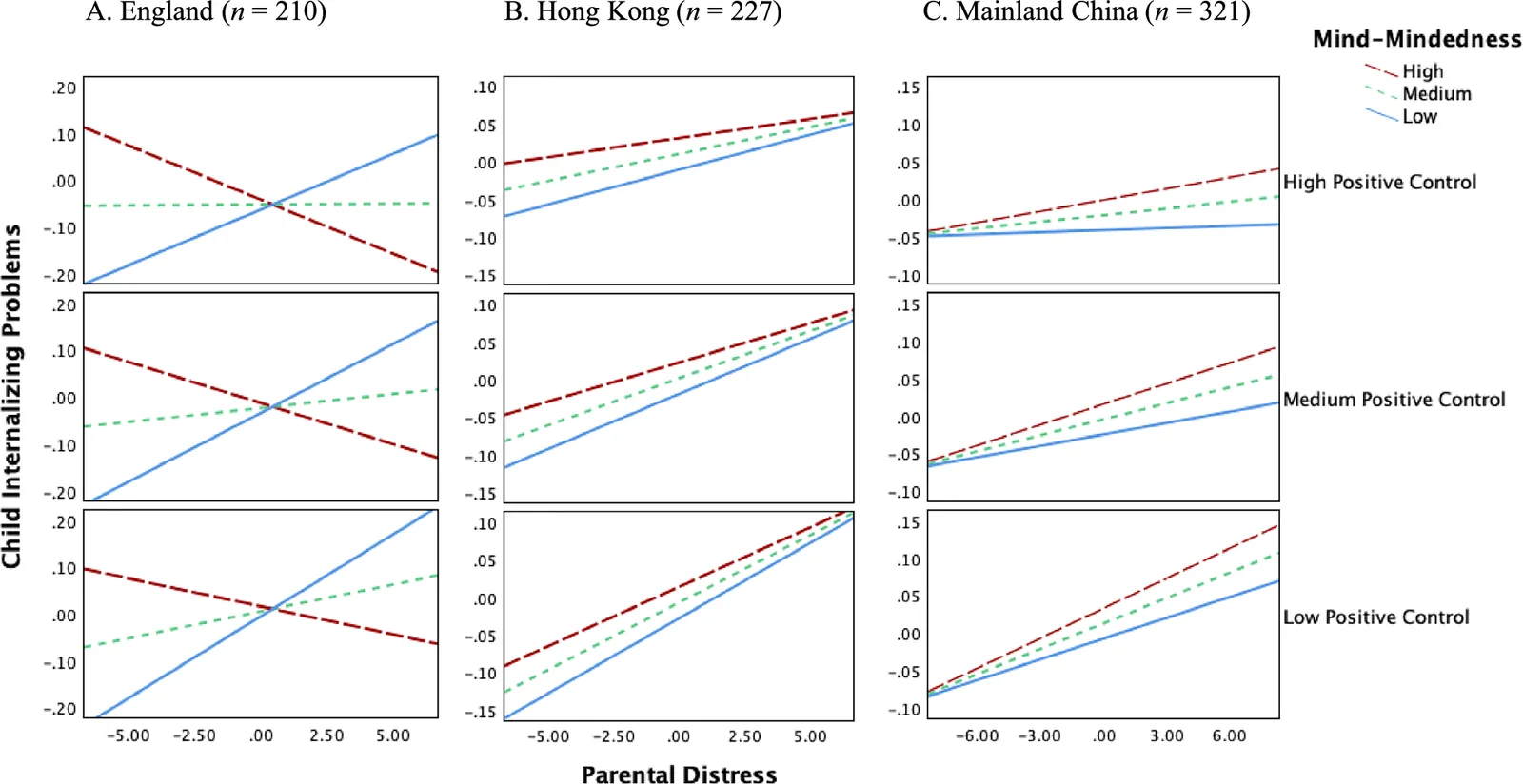
Visual representations of simple slopes illustrating the two-way interactions between parental distress and mind-mindedness, and between parental distress and positive control, by site*.* Panels **A**–**C** represent England, Hong Kong, and Mainland China, respectively. Within each panel, rows represent levels of positive control at low (−1 SD), medium (mean), and high (+1 SD). Lines represent model-estimated simple slopes of parental distress predicting child internalizing problems at low (−1 SD), medium (mean), and high (+1 SD) levels of mind-mindedness. Axis scales are site-specific. Each model included both interaction terms (mind-mindedness × distress; positive control × distress), allowing their effects to be estimated simultaneously. Formal simple slope tests were conducted only for statistically significant interaction effects; confidence intervals for all estimates are reported in Table S9.

We then probed these significant interactions using simple slopes. Figure 2 displays the predicted values of internalizing problems based on the conditional effects of parental distress at low (1 SD below the mean), medium (within 1 SD of the mean), and high (1 SD above the mean) levels of each moderator, with the other moderator held constant. In the English sample, parental psychological distress was significantly linked with child internalizing problems only at low levels of mind-mindedness, regardless of positive control. In Mainland China, parental distress was significantly associated with internalizing problems, but only at low and medium levels of positive control, regardless of mind-mindedness (see Table S9 for full results).

## Discussion

To address the narrow cultural scope of previous research into the processes linking parental psychological distress to child internalizing problems, our study included three study sites (i.e., England, Hong Kong, and Mainland China) characterized by contrasting norms regarding parenting goals and emotional expression^85^. We also included two direct parenting measures (i.e., positive control and mind-mindedness) and applied a meta-analytic approach to assess the across-site consistency of between-measure associations. Finally, we examined whether positive control and/or mind-mindedness attenuated links between parental distress and child internalizing problems in each site. Parental distress showed similar associations with child internalizing problems and parental positive control across sites, but culturally specific associations with mind-mindedness. The link between parental distress and child internalizing problems was attenuated in the context of high positive control in Mainland China, and in the context of average to high mind-mindedness in England. Multi-group modeling demonstrated site contrasts for the latter moderation effect. Below, we discuss these results in turn.

### Associations between study measures show both similarities and contrasts across sites

Our first single-study meta-analysis demonstrated a consistently positive association between parental distress and child internalizing problems, extending the evidence for intergenerational stability in internalizing problems beyond European and North American contexts of previous studies of clinical^86^ and community samples^20^. This cross-site consistency is notable, given cultural differences in norms regarding emotional expression and parenting^87^. In line with prior work^74^, we established partial scalar invariance for the Strengths and Difficulties Questionnaire’s internalizing subscale across sites, although this required the removal of four items. Similar item reductions have been reported in cross-cultural studies using the SDQ, often reflecting differences in item interpretation (e.g., reverse-worded items^88^) or context-specific influences (e.g., prolonged school closures during the pandemic^89^). These changes underscore the need for further cultural adaptation to enhance the cross-cultural applicability of measures of child adjustment. Strikingly, the inverse association between parental distress and positive control was also consistent across sites, despite significant across-site contrasts in mean levels of positive control (ENG > HK > MC). This pattern indicates the robustness of the parental distress–positive control link across cultural contexts and is consistent with prior work suggesting that within-culture variability in parenting may often exceed between-culture contrasts^55^.

That said, heterogeneity testing from our single-paper meta-analysis indicated that the association between parental distress and mind-mindedness did differ significantly across sites. As noted in the introduction, associations between parental distress and mind-mindedness appear attenuated in contexts of stress, and our data collection took place during the COVID-19 pandemic. Thus, the across-site heterogeneity may reflect site contrasts in the severity and/or duration of Covid-19 pandemic-related stressors (e.g., school closures). Future longitudinal work is needed to investigate parental mind-mindedness in the context of transient *versus* chronic distress. Another potentially fruitful research avenue might be to explore developmental contrasts, as prior investigations of links between parental distress and mind-mindedness have involved families with very young children^41–43^. Parental representations become consolidated with child age, and so the effects of depression or anxiety on mind-mindedness might be expected to diminish.

### Moderators of the link between parental distress and child internalizing problems differ across sites

In Mainland China, parental distress was only associated with child internalizing problems when parents showed average or low levels of positive control. That is, across all levels of parental mind-mindedness, the association between parental distress and child internalizing problems was non-significant in the context of high positive control. This indicates that positive control may be a useful focus for interventions to mitigate the emotional impact of exposure to parental distress.

As the magnitude of this moderation effect showed no significant site contrasts that might suggest culturally-specific influences, the null results in England and Hong Kong (both characterized by higher mean levels of positive control) may indicate that variability in positive control has limited influence on the association between parental distress and child internalizing problems, beyond a threshold ‘good enough’ level. Another plausible interpretation hinges on how positive control operates within the family context across cultures. Meta-analytic evidence synthesizing findings from 108 independent data sets across 22 cultures shows that collectivism strengthens the internalization of socially mediated experiences, amplifying their impact on psychological outcomes (e.g., the internalization of stigma^90^). By analogy, positive control in Mainland China may be more salient to children’s meaning-making processes, thereby more effectively buffering the impact of parental distress.

Future studies might test the ‘good enough’ threshold proposal by observing parent-child interactions across settings that evoke varying levels of positive control (e.g., goal-directed play *versus* free-play). It is also notable that, compared with parents in Mainland China, Hong Kong parents showed higher average levels of parental distress and positive control. Thus, parents’ high educational aspirations in Hong Kong^87^ may have enabled parents to offer praise, encouragement and autonomy support within this guided learning context, even in the presence of psychological distress. Note, however, that questionnaire-based studies indicate *higher* levels of authoritarianism among parents of 4th- and 5th-grade children in Hong Kong than in Mainland China^91^. This picture of mixed contrast across different methods of data collection (survey versus direct observations) suggests that the enduring legacy of British colonialism may have left residual effects on Hong Kong culture, alongside its clear cultural history of its own.

Evidence for cultural contrasts in parents’ cognitions also comes from a recent study of parental ethno-theories, often implicit, culturally informed belief systems, that involved 108 parents of 5-year-old children living in Mainland China or the USA^92^. Consistent with a ‘universality without uniformity’ model, the parents in this study showed both common and distinct concerns. Both groups of parents viewed social insensitivity as most worrying and were much less concerned about physical weakness. However, parental values also showed some between-group differences. First, child aggression and disruptive behavior emerged as key foci of concern for parents in the USA, while cognitive and motivational difficulties evoked greater concern among Chinese parents. Second, misbehavior was typically attributed to transient internal states by American parents, but to social factors or temperament by Chinese parents. Of relevance to our study’s focus on internalizing problems, cross-cultural contrasts have also been noted in relation to shyness in children. For example, in a study of families with 8–10-year-old children living in Canada (*n* = 149) or China (*n* = 145), Kong et al.^93^ distinguished between two forms of shyness (anxious and regulated) and showed that while anxious shyness showed a similar relationship with both depression and aggression in both sites, regulated shyness was only related to (low levels of) depression and aggression in Canadian children. This suggests that dispositional factors are differentially salient for Canadian children, whereas for Chinese children, regulated shyness is typically a response to external influences that promote cultural norms of modesty and harmony^94^. In future studies, supplementing measures of mind-mindedness and direct observations of positive control with assessments of parental ethno-theories may offer a promising avenue for exploring site contrasts in potential buffers that can break the intergenerational cycle of distress.

Turning to mind-mindedness, robust evidence for associations with sensitive caregiving^66^ might lead one to expect an attenuated association between parental distress and child internalizing problems in the context of high mind-mindedness. Indeed, for families in England, our findings suggested that even average levels of mind-mindedness appeared sufficient to limit the emotional impact of exposure to parental distress. Moreover, this moderation effect held true across all levels of positive control.

Notably, the moderation effect for mind-mindedness was specific to England, perhaps reflecting across-site contrasts in the associations between parental distress and mind-mindedness. Given that a proclivity to focus on mental states may reduce the likelihood of parents viewing child behaviors as irritating or difficult^58^, it may be that the strong emphasis on restraint of emotional expression within Confucian societies (including Hong Kong and Mainland China^95^) makes mind-mindedness superfluous in terms of limiting children’s exposure to parental negative affect.

### Strengths, limitations, and future directions

By recruiting a relatively large sample (*N* = 849 families) from three culturally distinct sites, the current study expands the scope of research on family influences on child adjustment beyond North America and Europe. Our focus on children making the transition into formal schooling also extends the developmental scope of the existing literature on positive control, mind-mindedness, and internalizing problems beyond its heavy focus on infants and toddlers. Additionally, our use of direct observations of parent–child interactions and transcript-based coding of parental mind-mindedness reduced the likelihood of desirability effects that can affect the reliability of survey-based data.

However, six study limitations deserve mention. First, gathering multiple measures of parent–child interaction quality would have enabled us to assess measurement invariance across sites and apply latent variable analyses. At the same time, concerns about burden to families (all seen during the pandemic) meant that we limited our observations to one brief shared drawing task. Second, we only included a single index of mentalizing; different findings may emerge from other measures (e.g., interactive mind-mindedness, reflective functioning) or from analyses that include the valence of mentalistic comments. For example, among Chinese children making the transition to siblinghood, positive trajectories of emotional adjustment were unrelated to overall mind-mindedness, but did appear associated with *positive* mind-mindedness, that is, the proportion of mentalistic child descriptions that were positive in valence^57^. Third, across all three sites, only a small percentage of fathers (5–11%) took part, despite study invitations including both fathers and mothers, and remote observations reducing the logistical challenges of participation. Recent meta-analytic findings indicate that, although fathers show less propensity to mentalize than mothers, within-couple associations are significant^96^. Thus, future studies need to adopt more strategic approaches to recruit fathers, both to test the generalizability of study findings and to examine the interplay between maternal and paternal influences. Fourth, as our analyses did not include any child behavior measures from the shared drawing task, the cultural universality of our study findings may at least partly reflect across-site similarities in child behavior. That said, preliminary findings from ongoing transcript-based analyses indicate that most children followed their parents’ lead in this online drawing task, indicating that in this goal-directed setting, child-driven effects may be quite small. Fifth, our study samples were ethnically relatively homogeneous within each site; future work is needed to integrate within- and between-site cultural contrasts in parenting. Sixth, we did not distinguish between different profiles of parent distress, despite evidence that different features of parental stress, depression, and anxiety have distinct links with parenting behaviors^97^. Exploring these differential links within a multi-site design would be a promising area for future research.

### Conclusions

Consistent with a universality without uniformity model of parental influences on child outcomes, across our three study sites, parental distress showed similar links with child internalizing problems and positive control, but contrasting links with mind-mindedness. Perhaps reflecting ‘good enough’ threshold effects, it was only in Mainland China, the site with the lowest average levels of positive control, that the association between parental distress and child internalizing problems was attenuated by high positive control. Conversely, the intergenerational link in internalizing problems was only attenuated by mind-mindedness in England, highlighting the need for more in-depth multi-measure studies of mentalizing as a potential protective factor in different settings. As highlighted by calls for a chronospatial revolution in psychology, it is vital to extend research into the determinants of childhood well-being in populations outside North America and Europe, who are almost completely overlooked in existing research efforts.

## Supplementary information

Transparent Peer Review file

Supplementary materials for

## Data Availability

The data necessary to reproduce the analyses presented here are publicly accessible; data are available at the following URL: https://osf.io/kfxuc.

## Code availability

The analytic code necessary to reproduce the analyses presented here is publicly accessible; syntax is available at the following URL: https://osf.io/kfxuc.
