# Supplementary material for: Cross-cultural evidence for positive control and mind-mindedness as buffers for parental distress
Source: Commun Psychol. 2026 Jun 17;4:133. doi: 10.1038/s44271-026-00484-x (PMC13634921; doi:10.1038/s44271-026-00484-x)
Supplement: Supplementary file 1 [file 44271_2026_484_MOESM1_ESM.pdf]

nature portfolio

Peer Review File

Cross-Cultural Evidence for Positive Control and Mind-Mindedness as Buffers for Parental Distress

Corresponding Author: Professor Claire Hughes

This file contains all editorial decision letters in order by version, followed by all author rebuttals in order by version.

Version 0:

Decision Letter:

** Please ensure you delete the link to your author homepage in this e-mail if you wish to forward it to your coauthors **

Dear Professor Hughes,

Thank you for your patience during the peer-review process; we apologize for the prolonged review process. Your manuscript titled "Raising resilience: Do positive parenting and mind-mindedness mitigate effects of exposure to parental distress?" has now been seen by 2 reviewers, and I include their comments at the end of this message. They find your work of interest but raised some important points. We are interested in the possibility of publishing your study in Communications Psychology, but would like to consider your responses to these concerns and assess a revised manuscript before we make a final decision on publication.

We therefore invite you to revise and resubmit your manuscript, along with a point-by-point response to the reviewers. Please highlight all changes in the manuscript text file.

Please clarify and unify all constructs, with particular attention to mind-mindedness and resilience, further justify the methodological decisions, improve figure clarity, and incorporate additional relevant literature.

As you revise the manuscript in response to these issues, please also implement all requests in the attached Mandatory Revision Requests document. All requirements listed in this document need to be fully met, or the work will be returned to you for further revisions without peer review. This workflow is in place to increase the likelihood that the paper will be accepted for publication. It reduces the number of rounds of revision (and review) and ensures that the reviewers vet a version of the article that is compliant with journal policies. If you have any questions regarding the required revisions, please contact the journal prior to resubmission to avoid a negative outcome.

We are committed to providing a fair and constructive peer-review process. Please don't hesitate to contact us if you wish to discuss the revision in more detail.

Please submit the following items:

- Revised manuscript
- Point-by-point response to the referees' comments
- Mandatory Revision Requests Table (attached).
- Cover letter (as a separate document)

via this link: Link Redacted .

** This url links to your confidential home page and associated information about manuscripts you may have submitted or are reviewing for us. If you wish to forward this email to co-authors, please delete the link to your homepage first **

We hope to receive your revised paper within 8 weeks; please let us know if you aren't able to submit it within this time so that we can discuss how best to proceed. If we don't hear from you, and the revision process takes significantly longer, we may close your file. In this event, we will still be happy to reconsider your paper at a later date, provided it still presents a significant contribution to the literature at that stage.

We look forward to seeing the revised manuscript and thank you for the opportunity to review your work.

Best regards,

Shir Atzil

Shir Atzil
External Editor
Communications Psychology

REVIEWER EXPERTISE:
Reviewer #1: Child development and regulation
Reviewer #2: Child development and resilience

REVIEWER REPORTS:

Reviewer #1 (Remarks to the Author):

This is an interesting study, with a rich data source and interesting questions. Although I applaud the authors efforts to examine cultural differences in factors that may help distress parenting in refraining from transmitting their distress to their children, I think that this manuscript would require substantial modifications before it is published. I therefore advise it to be rejected, but I hope that the authors are able to modify the manuscript and publish the results of this rich dataset.

Abstract: The abstract is difficult to follow if you haven't read the manuscript. For instance: what is representational mind-mindedness. Also please check sentence: "Within-site analyses showed that parent-child associations in internalizing "

The introduction is difficult to follow and fragmented. The authors should structure the introduction better and provide evidence for all their claims. Below, please find some suggestions for improvement, but in general I would advice the authors to critically go through the introduction to check whether hypotheses are well explained and supported by literature.
Introduction: can you give examples of " predict negative long-term outcomes"
In "For example, 17 of the 19 studies reviewed by Rose et al.19 54 55 took place in these regions." what was this review about?

In the paragraph about multisite findings, there can be improvements: for some studies, the authors don't give any information about which sites are examined. Potentially, they are all from Minority world countries. The authors also mention something about mind-mindedness in one of the studies, but this is not clear. Specifically, the authors argue that: "'mindful parenting' has culturally universal determinants". What is this conclusion based on? Then, the authors argue: "By contrast, in 62 a 9-site survey of 1,315 families with young adolescents, associations between parental 63 rejection and child adjustment problems were stronger in sites with higher normative levels of parental rejection22". As the rest of the paragraph is on the association between parental distress and child mental health, it is unclear how this study, that's on different concepts, fits with the other studies.
Please explain the 'universality without uniformity' perspective.
The authors use different concepts throughout the paper. They start with discussing parental distress, but then move to parental anxiety to explain why associations with parenting may be expected. But what is expected for parents scoring high on depression? Later (line 86), the authors only focus on depression. Provided these specific expectations for anxious and depressed parents, does it make sense to examine anxiety and depression together as distress?
The paragraph on self-determination theory could profit from more explanation. The authors use SDT to explain why certain parenting behaviors may result in child distress (this could actually also be more explicit) but then also make claims about parental distress predicting parenting behavior, without providing any references, e.g.: "With regards to relatedness, 74 parents who feel overwhelmed may struggle to respond to their child" Does SDT also explain the link from parental distress to parenting behaviors. And how?
In the paragraph Is parental distress related to reduced positive control and mind-mindedness?, the authors do not explain if parental distress is related to positive control. In the general, this section is difficult to follow, as it has all kinds of argumentation that seems irrelevant.
It is unclear why the theory of Grolnick and Pomerantz is outlined in the section about moderator effects. It is also unclear how Grolnick and Pomerants' theory relates to the concepts used in this study. Positive control is framed as the authors as

autonomy support, which does not map on structuring, but rather reflects the positive end of the other dimension that Grolnick and Pomerantz propose.
The manuscript makes many claims without references. E.g., For example, parental control is traditionally associated with care and concern within East Asian cultures, but with hostility in Western cultures.
"we anticipated that positive control in this drawing task would be especially salient.."What do the authors mean with salient? For internalizing problems in children? Or just that it is more prevalent? And what is the argumentation behind this expectation?
In methods:
Parental positive control: I appreciate the use of behavioral observations. However, I wonder to what extent this concerns control. Why is asking open ended questions and praising a form of positive control?
Parental mind-mindedness: can the authors explain what the five-minute speech sample paradigm entailed?
In "Accordingly, we indexed mind-mindedness by the frequency of mental attributes, covarying the frequency of non mental attributes to account for parental verbosity" what is meant by "covarying"? Can the authors explain the procedure to come to a score?
Parental distress: what type of score was created? Were all scores added together?
Child internalizing problems: Why is peer problems considered as internalizing problems? This is also not in line with the definition provided by authors.
In Table 1, what is the difference between degree and social ladder? And how do participants in MC more often have a high degree but a lower average score on the social ladder?
Results
Why is parental distress estimated by loading the total scores on a factor, whereas child internalizing problems is based on a factor scores on the items? I would make this consistent.
Was Measurement invariance tested across sites?
The association between parental distress and parental mind-mindedness is only discussed for England, why not for the other sites? (line 289)
I would consider conducting the meta-analysis on the regression coefficients instead of the correlations. Now, much of the results is based on correlations, but the authors can also control for confounders and then conduct a meta-analysis.
In Table 4, please put the control variables on top, next the main effects and then the moderators.
The authors find a significant moderator in England (distress* mind mindedness) and China (distress* control), but only visualize the simple slopes for positive control. Please only visualize the slopes for significant interactions, and also include the simple slope for distress* mind-mindedness for England.

I wonder to what extent mind-mindedness is a cultural phenomenon that fits with the way in which Minority world parents raise their children – the high mean for English parents gives an indication that this may just not be the way in which non-minority world parents parent their children.

Reviewer #2 (Remarks to the Author):

The article titled "Raising Resilience: Do positive parenting and mind-mindedness mitigate effects of exposure to parental distress" was a pleasure to read. The authors aimed to identify cross-context associations between positive control and parent mind mindedness on buffering the association between parental psychological distress and child internalizing problems across cultural contexts. The study is well powered, and the authors were thoughtful about measurement invariance assessments in cross-context comparisons of results. The authors also utilized multiple methods to assess study constructs. This will clearly be a great contribution to the literature on cross-cultural parenting behaviors and their contextually specific effects on child mental health. I raise the following mostly minor suggestions with the goal of strengthening the manuscripts contribution.

1) The authors should consider changing the title. The authors do not thoroughly discuss the concept of resilience in the text of the manuscript and instead appear to be discussing risk for psychopathology. If authors wish to maintain resilience framing, they should consider a more explicit discussion of resilience theory in the manuscript.
2) The manuscript would benefit from incorporating a few additional literatures into the introduction and discussion of results:
a. The authors very minimally discuss the role of child age in the current analyses. Please consider describing the developmental implications of studying internalizing disorders in preschool/early elementary aged children. Please also consider the specific developmental role of these specific parenting strategies at this age.
b. The authors importantly discuss the importance of studying the study constructs in non-western countries. It would be beneficial if the authors elaborated more on how they expect these behaviors to vary based on cultural ideals and values. What specific aspects of each context may shift parenting values/goals/behaviors? How might future studies do a better job of capturing within context variation in these cultural attributes/values/beliefs that may influence these differences in associations?
3) Do the authors have available information about participant race/ethnicity across study sites? Although they are living in specific countries/regions they could still vary by racial/ethnic makeup or history which can also shift cultural expectations and practices in parenting.
4) Please provide more information about your education and social ladder measure.
5) On line 416 the authors describe "positive mind-mindedness" please further define or describe this construct.
6) In the discussion, the authors should re-emphasize the limitations in internalizing disorder measurement in some of the study contexts.
7) Figure 2 is a bit challenging to follow. Perhaps consider relabeling to make it clear what the context of the stacked graph

is. If possible, reduce use of acronyms and potentially find a way to denote what are significant differences based on the simple slopes analysis.
a. Did the authors conduct a three-way interaction? If not, please consider showing the two-way interaction only in separate graphs. If you did, please report the three-way interaction in Table 4.

* TRANSPARENT PEER REVIEW: Communications Psychology uses a transparent peer review system. This means that we publish the editorial decision letters including Reviewers' comments to the authors and the author rebuttal letters online as a supplementary peer review file. However, on author request, confidential information and data can be removed from the published reviewer reports and rebuttal letters prior to publication. If your manuscript has been previously reviewed at another journal, those Reviewers' comments would not form part of the published peer review file.

** Visit Nature Research's author and referees' website at www.nature.com/authors for information about policies, services and author benefits**

Communications Psychology is committed to improving transparency in authorship. As part of our efforts in this direction, we are now requesting that all authors identified as 'corresponding author' create and link their Open Researcher and Contributor Identifier (ORCID) with their account on the Manuscript Tracking System prior to acceptance. ORCID helps the scientific community achieve unambiguous attribution of all scholarly contributions. You can create and link your ORCID from the home page of the Manuscript Tracking System by clicking on 'Modify my Springer Nature account' and following the instructions in the link below. Please also inform all co-authors that they can add their ORCIDs to their accounts and that they must do so prior to acceptance.
https://www.springernature.com/gp/researchers/orcid/orcid-for-nature-research

For more information please visit http://www.springernature.com/orcid

If you experience problems in linking your ORCID, please contact the Platform Support Helpdesk.

Version 1:

Decision Letter:

** Please ensure you delete the link to your author homepage in this e-mail if you wish to forward it to your coauthors **

Dear Professor Hughes,

Your manuscript titled "Breaking the Cycle of Distress via Buffering Effects of Positive Control and Mind-Mindedness" has now been seen by our reviewers, whose comments appear below. In light of their advice I am delighted to say that we are happy, in principle, to publish a suitably revised version in Communications Psychology.

We therefore invite you to revise your paper one last time to address the remaining concerns of our reviewers and a list of editorial requests. At the same time we ask that you edit your manuscript to comply with our format requirements and to maximise the accessibility and therefore the impact of your work.

EDITORIAL REQUESTS:

Please review our specific editorial comments and requests regarding your manuscript in the attached "Editorial Requests Table". Please outline your response to each request in the right hand column. Please upload the completed table with your manuscript files as a Related Manuscript file.

If you have any questions or concerns about any of our requests, please do not hesitate to contact me.

SUBMISSION INFORMATION:

In order to accept your paper, we require the files listed here https://www.nature.com/documents/commsj-file-checklist.pdf .

OPEN ACCESS:

Communications Psychology is a fully open access journal. Articles are made freely accessible on publication. For further information about article processing charges, open access funding, and advice and support from Nature Research, please visit https://www.nature.com/commspsychol/open-access

At acceptance, you will be provided with instructions for completing the open access licence agreement on behalf of all authors. This grants us the necessary permissions to publish your paper. Additionally, you will be asked to declare that all

required third party permissions have been obtained, and to provide billing information in order to pay the article-processing charge (APC).

* TRANSPARENT PEER REVIEW: Communications Psychology uses a transparent peer review system. On author request, confidential information and data can be removed from the published reviewer reports and rebuttal letters prior to publication. If you are concerned about the release of confidential data, please let us know specifically what information you would like to have removed. Please note that we cannot incorporate redactions for any other reasons.

* CODE AVAILABILITY: All Communications Psychology manuscripts must include a section titled "Code Availability" at the end of the methods section. We require that the custom analysis code supporting your conclusions is made available in a publicly accessible repository at this stage; please choose a repository that generates a digital object identifier (DOI) for the code; the link to the repository and the DOI must be included in the Code Availability statement. Publication as Supplementary Information will not suffice.

* DATA AVAILABILITY:
All Communications Psychology manuscripts must include a section titled "Data Availability" at the end of the Methods section. More information on this policy, is available in the Editorial Requests Table and at http://www.nature.com/authors/policies/data/data-availability-statements-data-citations.pdf.

Please use the following link to submit the above items:
Link Redacted
** This url links to your confidential home page and associated information about manuscripts you may have submitted or be reviewing for us. If you wish to forward this email to co-authors, please delete the link to your homepage first **

We hope to hear from you within two weeks; please let us know if you need more time.

Best regards,

Jennifer Bellingtier

Jennifer Bellingtier, PhD
Senior Editor
Communications Psychology

Shir Atzil
External Editor
Communications Psychology

REVIEWER EXPERTISE:

Reviewer #2: Child development and resilience

REVIEWERS' COMMENTS:

Reviewer #2 (Remarks to the Author):

The reviewer assessed a revision of the article now titled “Breaking the Cycle of Distress via Buffering Effects of Positive Control and Mind-Mindedness”. The authors aimed to identify cross-context associations between positive control and parent mind mindedness on buffering the association between parent distress and child internalizing problems across cultural contexts. The authors were very responsive to reviewer comments and have strengthened the manuscript. I raise the following suggestions to further strengthen the author’s contribution:
1) In the abstract the authors define positive control as autonomy support. This could use a more specific definition about what autonomy support is or manifests as in measurement/observation.
2) In the introduction on page 2 the authors discuss “parental buffers” but are not clear about what parenting is buffering. Please consider clarifying
3) At the top of page 4 the authors report “the relative salience of contextual effects” but it is unclear what they are referring to in that sentence
4) On page 7 the authors report that “parental depression was only associated with reduced mind-mindedness if infants show low levels of distress”, and “parenting stress was associated with low mind mindedness in the context of high social

support”. The authors interpret this finding as mind-mindedness as being difficult in times of stress/parent internalizing symptoms. To me it suggests that in the context of stressful circumstances (e.g., high infant distress or low social support), that parent internalizing symptoms of distress was not associated with mind-mindedness. The findings as presented do not allow us to infer anything about how circumstances of stress are related to mind-mindedness abilities. Can the authors please clarify these findings and descriptions in the text. Was there a main effect of the “stress” circumstance on mind-mindedness?
a. For this paper’s analyses can the authors confirm in text if there was any evaluation or main effect of COVID-19 stress exposure on level of mind-mindedness?
5) Page 9 the authors refer to “basic psychological needs model”. Please define or explain this model.
6) On page 19 the authors interpret the association between parental distress and positive control as being significant across site as evidence for more within-culture variability than between culture variability. I am not sure this analysis points to that exact evidence. That sort of question may be better answered by looking at spread and variability in the data. This question may also be answered by running a multilevel model with ICC values or random-effect ANOVA for example, to evaluate if between or within country variance in parenting.
7) On page 20 the authors report that positive control only buffered the effects of parent distress on child internalizing symptoms in mainland China. This is interesting given that the effect sizes appear to be equivalent across sites. The authors astutely point towards average level differences in positive control across sites with Mainland China being almost 2 points lower on average on this assessment. I appreciate the “good enough” positive control/parenting interpretation, but given the cross-cultural evaluation goals of this study, I wonder if the authors have considered any cultural differences in Mainland China that may play a role in these findings.
8) On page 21 the authors describe different parenting concerns of priorities for children’s behaviors. Interestingly, most of these examples would fall under concerns for externalizing behaviors. I wonder if there are any studies about parenting values for internalizing sorts of behaviors?
9) When discussing mind-mindedness findings in the discussion, the authors do not provide an explanation about site-based differences that may lead to differences in association. Please consider adding.
10) For all variables, were there main effects of mind-mindedness, positive control, and parent psychological distress on child internalizing outcomes? Did you assess these models WITHOUT interactions and then assess if model fit improved with the inclusion of moderation effects?
11) One limitation of the study may be that all samples were made up primarily by the majority race/ethnicity in their country. This may include an expanded discussion around how within country culture can vary and may lead to heterogeneity within associations both within and across countries

** Visit Nature Research's author and referees' website at www.nature.com/authors for information about policies, services and author benefits**

Open Access This Peer Review File is licensed under a Creative Commons Attribution 4.0 International License, which permits use, sharing, adaptation, distribution and reproduction in any medium or format, as long as you give appropriate

credit to the original author(s) and the source, provide a link to the Creative Commons license, and indicate if changes were made.
In cases where reviewers are anonymous, credit should be given to 'Anonymous Referee' and the source.
The images or other third party material in this Peer Review File are included in the article's Creative Commons license, unless indicated otherwise in a credit line to the material. If material is not included in the article's Creative Commons license and your intended use is not permitted by statutory regulation or exceeds the permitted use, you will need to obtain permission directly from the copyright holder.
To view a copy of this license, visit https://creativecommons.org/licenses/by/4.0/

**Associate Editor**
Thank you for your patience during the peer-review process; we apologize for the prolonged review process. Your manuscript titled "Raising resilience: Do positive parenting and mind-mindedness mitigate effects of exposure to parental distress?" has now been seen by 2 reviewers, and I include their comments at the end of this message. They find your work of interest but raised some important points. We are interested in the possibility of publishing your study in *Communications Psychology*, but would like to consider your responses to these concerns and assess a revised manuscript before we make a final decision on publication. Please clarify and unify all constructs, with particular attention to mind-mindedness and resilience, further justify the methodological decisions, improve figure clarity, and incorporate additional relevant literature.
Response:<br>Thank you very much for your careful consideration of our manuscript (COMMSPSYCHOL-25-0722-T) and for coordinating the review process. We'd also like to thank the reviewers for their thoughtful and constructive feedback. The manuscript was initially submitted to *Nature Communications*, which has strict page and reference limits (no more than 35 pages and 70 references). We therefore appreciate the opportunity to revise the manuscript and improve its clarity by adding more information about measures / constructs and by providing more references to back up the claims made. Below we provide a detailed, point-by-point response to the reviewers' comments, indicating how each concern has been addressed and where corresponding revisions have been made in the manuscript. We are grateful to both reviewers, as the requested changes have strengthened the manuscript considerably.<br><br>In addition, we have addressed the editorial comments in the Editorial Request Table; corresponding revisions in the manuscript and Supplementary Materials are listed below:<br>▪ Page 2 (lines 16–17)<br>▪ Page 4 (lines 68–70)<br>▪ Page 8 (line 170)<br>▪ Page 9 (lines 192–194)<br>▪ Page 10 (lines 228–229, 238–239)<br>▪ Page 11 (lines 247–248)<br>▪ Pages 14–15 (lines 339–341)<br>▪ Page 15 (lines 355–356, 360)<br>▪ Page 16 (lines 367–368, 371–372, 385–388)<br>▪ Page 17 (line 396)<br>▪ Page 18 (lines 414–415, lines 417–418)<br>▪ Page 23 (lines 551, 560)<br>▪ Page 25, Table 1<br>▪ Page 29, Table 4,<br>▪ Page 30, Figure 2,<br>▪ Page 31, Data Availability Statement, Code Availability Statement<br>▪ Page 45, Conflict of Interest Statement, Acknowledgements<br>▪ Supplementary Materials, Page 7, Tables S4–S5<br>▪ Supplementary Materials, Pages 8–10, Table S6

**Reviewer 1**
Abstract: The abstract is difficult to follow. For instance: what is representational mind-mindedness. Also please check sentence: "Within-site analyses showed that parent-child associations in internalizing."
Response:

Apologies for the ambiguity – we have now added the term "**representational**" before the definition given for mind-mindedness at the start of the abstract. We have also replaced the phrase "**parent-child associations in internalizing**" with the phrase "**intergenerational associations in distress-internalizing problems,**" which we hope is clearer.<br><br>Revision in manuscript:<br>▪ Page 1 (lines 4–5, 7–12)
The introduction is difficult to follow and fragmented. The authors should structure the introduction better and provide evidence for all their claims. Below, please find some suggestions for improvement, but in general I would advise the authors to critically go through the introduction to check whether hypotheses are well explained and supported by literature. Introduction: Can you give examples of "predict negative long-term outcomes"?
Response:<br>Thank you for your helpful suggestions. As noted above, our original manuscript was constrained by space limits, but we have now added a few lines to clarify the structure of our introduction: "**This introductory section is structured around three questions. In the first and second sections, we ask whether, across cultures, parental distress is linked with (a) child internalizing problems, and (b) reduced positive control and mind-mindedness. Third, we ask whether positive control and parental mind-mindedness attenuate the association between parental distress and child internalizing problems.**"<br><br>We have also added **21 new references.** Regarding this specific point in the introduction, we have added the phrase "**such as mood disorders** (Angold & Egger, 2007)," which we hope adds clarity.<br><br>Revision in manuscript:<br>▪ Page 2 (line 20)<br>▪ Page 4 (lines 70–74)
In "For example, 17 of the 19 studies reviewed by Rose et al. took place in these regions." What was this review about?
Response:<br>To clarify, the text now reads: "**However, existing evidence is very limited in geographical scope. For example, of the 19 studies included in Rose et al.'s (2018) systematic review of links between parenting and child internalizing problems, 17 involved samples from North America, Europe or Australia.**"<br><br>Revision in manuscript:<br>▪ Page 4 (lines 85–88)
In the paragraph about multisite findings, there can be improvements: for some studies, the authors don't give any information about which sites are examined. Potentially, they are all from Minority world countries.
Response:<br>Thank you for spotting this omission, which we have tried to rectify throughout. For example, we have now added details about the specific sites being examined in the relevant studies: "**a survey-based study of 17-year-olds (*N* = 12,757) from ten cultures (China, Czech Republic, Hungary, the Netherlands, Slovenia, Spain, Switzerland, Taiwan, Turkey, and the United States)… (Vazsonyi et al., 2022)**" and "**another 9-site survey-based study of 1,315 families with young adolescents (China, Colombia, Italy, Jordan, Kenya, the Philippines, Sweden, Thailand, and the United States)… (Rothenberg et al., 2022)**".<br><br>Revision in manuscript:<br>▪ Pages 4 (lines 87–88, 90)

- Page 5 (lines 93–100, 102, 113)
- Page 6 (lines 134–135)
- Page 7 (lines 143–145, 162– 163)

The authors also mention something about mind-mindedness in one of the studies, but this is not clear. Specifically, the authors argue that: "'mindful parenting' has culturally universal determinants". What is this conclusion based on?

Response:

We have re-written the text to clarify that this conclusion echoes the views expressed by others. The revised sentence now reads: "**In a study of 173 families with young adolescents from the UK or Türkiye, associations between parental distress and child internalizing problems were similar across sites, leading the authors to conclude that 'mindful parenting' is 'culture-free' (Acet et al., 2024).**"

Revision in manuscript:

- Page 4–5 (lines 90–92)

Then, the authors argue: "By contrast, in a 9-site survey of 1,315 families with young adolescents, associations between parental rejection and child adjustment problems were stronger in sites with higher normative levels of parental rejection (Rothenberg et al., 2022)". As the rest of the paragraph is on the association between parental distress and child mental health, it is unclear how this study, that's on different concepts, fits with the other studies.

Response:

To clarify the relevance of the findings on rejection, we have added and revised the following explanatory text: "**Highlighting the relevance of this reported moderation effect for the current study, recent findings from a longitudinal study of 841 Pakistani mother-toddler dyads shows that harsh parenting (closely related to rejection) mediates the link between maternal depression and children's socio-emotional adjustment (Frost et al., 2025). Together, these findings support the universality-without-uniformity perspective (Soenens et al., 2015), in which common patterns in parenting are expected across sites, but with specific processes showing differing relative salience.**"

New references:

- Frost, A., Scherer, E., Chung, E. O., Gallis, J. A., Sanborn, K., Zhou, Y., ... & Maselko, J. (2025). Longitudinal pathways between maternal depression, parenting behaviors, and early childhood development: A mediation analysis. *International Journal of Behavioral Development*, *49*(3), 263–276. https://doi.org/10.1177/01650254251327372
- Soenens, B., Deci, E. L., & Vansteenkiste, M. (2017). How parents contribute to children's psychological health: The critical role of psychological need support. In *Development of self-determination through the life-course* (pp. 171–187). Dordrecht: Springer Netherlands. https://doi.org/10.1007/978-94-024-1042-6_13

Revision in manuscript:

- Page 5 (lines 100–106)

Please explain the 'universality without uniformity' perspective.

Response:

To clarify we have added the following explanatory texts:

- Page 3 (lines 45–49)

"**That is, core parental aspects may be widely shared – but vary in both function (e.g., the same behavior, such as vocalization, can promote either interdependence or independent exploration). There can also be site differences in the form of parental behaviors. For example, affection can be displayed physically via hugs / kisses or more indirectly, via educational support.**"

▪ Page 5 (lines 105–106)<br>"**… in which common patterns in parenting are expected across sites, but with specific processes showing differing relative salience.**"
The authors use different concepts throughout the paper. They start with discussing parental distress but then move to parental anxiety to explain why associations with parenting may be expected. But what is expected for parents scoring high on depression? Later (line 86), the authors only focus on depression. Provided these specific expectations for anxious and depressed parents, does it make sense to examine anxiety and depression together as distress?
Response:<br>We have added the following clarification: "**While adult anxiety and depression are often studied separately, there is growing support for an alternative dimensional approach that includes each of these indicators of parental distress (Snyder et al., 2023).**"<br><br>New reference:<br>- Snyder, H. R., Silton, R. L., Hankin, B. L., Smolker, H. R., Kaiser, R. H., Banich, M. T., ... & Heller, W. (2023). The dimensional structure of internalizing psychopathology: Relation to diagnostic categories. *Clinical Psychological Science*, *11*(6), 1044–1063. https://doi.org/10.1177/21677026221119483<br><br>Revision in manuscript:<br>▪ Page 4 (lines 81–83)
The paragraph on self-determination theory could profit from more explanation. The authors use SDT to explain why certain parenting behaviors may result in child distress (this could actually also be more explicit) but then also make claims about parental distress predicting parenting behavior, without providing any references, e.g.: "With regards to relatedness, parents who feel overwhelmed may struggle to respond to their child". Does SDT also explain the link from parental distress to parenting behaviors. And how?
Response:<br>To clarify, we have added and revised the following text, plus references for each of the subsequent examples given in this paragraph: "**Although not originally a developmental theory, self-determination theory has proved useful in parenting research (Joussemet et al., 2008). Examples of empirical support for this perspective come from evidence that (a) prenatal need satisfaction predicts Belgian mothers' postnatal wellbeing (Brenning & Soenens, 2017) and (b) parental distress constrains parental support for these basic psychological needs. For example, anxious parents who engage in over-controlling behavior to reduce uncertainty may constrain child autonomy, a pattern reflected in research on links between 'helicopter parenting' and youth anxiety and depression (Vigdal & Brønnick, 2022). Likewise, if anxious parents model avoidance behaviors, children may build a sense of the world as threatening, depleting their sense of themselves as competent (Soenens et al., 2017). And parents who feel overwhelmed may struggle to respond to their child, reducing children's sense of connectedness. Together, these pathways underscore potentially universal benefits of positive control (i.e., autonomy support) and mind-mindedness (i.e., attunement to child's inner states) for limiting the emotional impact of exposure to parental distress.**"<br><br>New references:<br>- Brenning, K., & Soenens, B. (2017). A self-determination theory perspective on postpartum depressive symptoms and early parenting behaviors. *Journal of Clinical Psychology*, *73*(12), 1729–1743. https://doi.org/10.1002/jclp.22480<br>- Joussemet, M., Landry, R., & Koestner, R. (2008). A self-determination theory perspective on parenting. *Canadian Psychology / Psychologie Canadienne*, *49*(3), 194–200.

- Sadoughi, M. (2024). Overparenting and adolescent's trait anxiety: Unraveling the roles of basic psychological needs frustration and emotion dysregulation. *Acta Psychologica*, *251*, 104579. https://doi.org/10.1016/j.actpsy.2024.104579
- Soenens, B., Deci, E. L., & Vansteenkiste, M. (2017). How parents contribute to children's psychological health: The critical role of psychological need support. In *Development of self-determination through the life-course* (pp. 171–187). Dordrecht: Springer Netherlands. https://doi.org/10.1007/978-94-024-1042-6_13
- Vigdal, J. S., & Brønnick, K. K. (2022). A systematic review of "helicopter parenting" and its relationship with anxiety and depression. *Frontiers in Psychology*, *13*, 872981. https://doi.org/10.3389/fpsyg.2022.872981

Revision in manuscript:

- Pages 5–6 (lines 110–124)

In the paragraph "Is parental distress related to reduced positive control and mind-mindedness?", the authors do not explain if parental distress is related to positive control. In the general, this section is difficult to follow, as it has all kinds of argumentation that seems irrelevant.

Response:

We agree that the link between parental distress and positive control was implied rather than explicitly stated in this paragraph. We apologize for this oversight and have now added the text shown in bold in the passage below: "This multi-path model supports a differentiated model of parenting in which protection, control and guided learning represent three key dimensions (Davies & Sturge-Apple, 2024) **– each of which are likely to contribute to parental use of positive control. Longitudinal evidence from American (Hare et al., 2024) and British (Psychogiou et al., 2017) samples confirms that parental distress constrains positive parenting.**"

New references:

- Hare, M. M., Feeney, K. E., & Parent, J. (2024). Longitudinal pathways from parent internalizing symptoms to parent and youth emotion functioning. *Child Psychiatry & Human Development*, *55*(5), 1211–1224. https://doi.org/10.1007/s10578-022-01482-w
- Psychogiou, L., Moberly, N. J., Parry, E., Nath, S., Kallitsoglou, A., & Russell, G. (2017). Parental depressive symptoms, children's emotional and behavioural problems, and parents' expressed emotion – Critical and positive comments. *PloS One*, *12*(10), e0183546. https://doi.org/10.1371/journal.pone.0183546

Revision in manuscript:

- Page 6 (lines 133–136)

It is unclear why the theory of Grolnick and Pomerantz is outlined in the section about moderator effects. It is also unclear how Grolnick and Pomerantz's theory relates to the concepts used in this study. Positive control is framed as the authors as autonomy support, which does not map on structuring, but rather reflects the positive end of the other dimension that Grolnick and Pomerantz propose.

Response:

We have added and revised the text shown in bold to the passage the Reviewer is referring to: "According to Grolnick and Pomerantz (2009), **capturing the complexity of parental socialization requires going beyond the traditional view of control as authoritarianism to adopt a multiple-forms approach that encompasses positive aspects of control that can facilitate children's competence. One example is structuring, which has been reported to underpin moderating effects of neighborhood risk on the impact of parental restrictions (Coley & Hoffman, 1996). Structuring is also known as 'behavioral control'(Soenens et al., 2017), which overlaps with the construct of 'positive control' (i.e., using praise and encouragement to guide child activities and maintain task focus). Expanding this prior work on within-culture contextual contrasts, we will compare whether mitigating effects of positive control vary across study sites.**"

Revision in manuscript:

- Page 7 (lines 152–161)

The manuscript makes many claims without references. For example, parental control is traditionally associated with care and concern within East Asian cultures, but with hostility in Western cultures.

Response:

We agree that the literature review in the initial submission was constrained by the reference limits required for *Nature Communications*. For the study below we have added the following reference:

- Deater-Deckard, K., Lansford, J. E., Malone, P. S., Alampay, L. P., Sorbring, E., Bacchini, D., ... & Al-Hassan, S. M. (2011). The association between parental warmth and control in thirteen cultural groups. *Journal of Family Psychology*, *25*(5), 790–794. https://doi.org/10.1037/a0025120

Elsewhere, we have also ensured that throughout the manuscript, statements are supported by appropriate references. Newly added references are highlighted in blue in both the main text and the reference list.

Revision in manuscript:

- Page 7 (line 165)

"We anticipated that positive control in this drawing task would be especially salient…" What do the authors mean with salient? For internalizing problems in children? Or just that it is more prevalent? And what is the argumentation behind this expectation?

Response:

We have clarified the text, which now reads: "**As parental involvement in child activities is a primary expression of care in Mainland China, we anticipated that positive control would play an especially salient role in reducing the risk for internalizing problems in this site.**" We hope that framing this prediction in terms of the 'universality without uniformity' perspective helps to clarify why we expected positive control to be a stronger moderator in Mainland China, where mothers may be especially likely to express care by supporting goal-directed activities.

Revision in manuscript:

- Page 8 (lines 170–173)
- See also new reference (Page 8, line 178)

New references:

- Wu, J., & Chentsova-Dutton, Y. (2026). Why the West minds and the East behaves: An integrative review of the cultural evolution of mind–behavior orientations. *Journal of Cross-Cultural Psychology*, *57*(1), 3–50. https://doi.org/10.1177/00220221251362805

In methods:
Parental positive control: I appreciate the use of behavioral observations. However, I wonder to what extent this concerns control. Why is asking open ended questions and praising a form of positive control?

Response:

We have added a brief explanation in the introduction: "**(i.e., using praise and encouragement to guide child activities and maintain task focus).**"And in the method, we have added: "**In this challenging task, parental positive control (e.g., use of praise, explanations, and open-ended questions) was rated on a 7-point scale.**" We hope that these two changes clarify why, in this challenging task context, praise and encouragement represent positive forms of control.

Revision in manuscript:

- Page 7 (lines 158–159)
- Page 11 (lines 254–255)

Parental mind-mindedness: can the authors explain what the five-minute speech sample paradigm entailed?
Response:<br>Thank you for raising this point. To clarify what the Five-Minute Speech Sample paradigm entails, we have added a more detailed description in the Methods section. The revised text now reads: "Parents described their child and their relationship with the child via the Five-Minute Speech Sample paradigm (Magaña et al., 1986). **Parents were given five minutes to describe their child and how they get along. They were instructed: "I'd like to hear your thoughts and feelings about [CHILD], in your own words. Please speak for five minutes, telling me what kind of a person [CHILD] is and how the two of you get along." If parents stopped speaking before the five-minute point, they were given a standardized prompt ("You have another X minutes. Can you say anything else about [CHILD]?"). Originally developed for clinical studies, the Five-Minute Speech Sample paradigm is now widely used in family research as a simple and effective method for capturing parents' thoughts and feelings about their child (Sher-Censor, 2015). Transcripts can be coded for a range of parental representations, including narrative coherence (Sher-Censor & Yates, 2015), family affective attitudes (Bullock & Dishion, 2007), and aspects of parental mentalizing such as reflective functioning (Adkins et al., 2018) and mind-mindedness (Hughes et al., 2018).**"<br><br>New references:<br>- Adkins, T., Luyten, P., & Fonagy, P. (2018). Development and preliminary evaluation of family minds: A mentalization-based psychoeducation program for foster parents. *Journal of Child and Family Studies*, *27*, 2519–2532. https://doi.org/10.1007/s10826-018-1080-x<br>- Bullock, B. M., & Dishion, T. J. (2007). Family processes and adolescent problem behavior: Integrating relationship narratives into understanding development and change. *Journal of the American Academy of Child & Adolescent Psychiatry*, *46*(3), 396–407. https://doi.org/10.1097/chi.0b013e31802d0b27<br>- Sher-Censor, E. (2015). Five Minute Speech Sample in developmental research: A review. *Developmental Review*, *36*, 127–155. https://doi.org/10.1016/j.dr.2015.01.005<br>- Sher-Censor, E., & Yates, T. M. (2015). Mothers' expressed emotion and narrative coherence: Associations with preschoolers' behavior problems in a multiethnic sample. *Journal of Child and Family Studies*, *24*(5), 1392–1405. https://doi.org/10.1007/s10826-014-9946-z<br><br>Revision in manuscript:<br>▪ Pages 11–12 (lines 259–271)
In "Accordingly, we indexed mind-mindedness by the frequency of mental attributes, covarying the frequency of non -mental attributes to account for parental verbosity" what is meant by "covarying"? Can the authors explain the procedure to come to a score?
Response:<br>Apologies for the confusion. We have revised the text to clarify this point. Specifically, the frequency of non-mental attributes was included as a covariate in subsequent analyses. The revised sentence now reads: "**Accordingly, we used the frequency count of mental attributes in parents' descriptions of their child to index mind-mindedness. Additionally, as in previous studies (e.g., Hughes et al., 2018), we accounted for parental verbosity by including the frequency of non-mental attributes as a covariate in subsequent analyses.**"<br><br>Revision in manuscript:<br>▪ Page 12 (lines 280–284)
Parental distress: what type of score was created? Were all scores added together?<br>In results:<br>Why is parental distress estimated by loading the total scores on a factor, whereas child internalizing problems is based on a factor scores on the items? I would make this consistent.

Response:

We address these two comments together, as both concern how parental distress was scored.

Parental distress was assessed using three well-validated measures capturing different aspects of distress: the Kessler Psychological Distress Scale (K10; 10 items), the Patient Health Questionnaire (PHQ-9; 9 items), and the Generalized Anxiety Disorder Scale (GAD-7; 7 items), yielding 26 items in total. As a first step, we examined measurement invariance separately for each scale across the English, Hong Kong, and Mainland China sites by specifying measurement models in which the items for each scale loaded onto their respective latent factors. These analyses established partial scalar invariance for each measure (see Pages 2–3 and Table S1 in the Supplementary Materials).

For the main analyses, to simplify the model structure, we specified a just-identified measurement model in which the total scores of the K10, PHQ-9, and GAD-7 loaded onto a single latent factor representing parental distress. Factor scores from this model were then used in subsequent analyses. To improve clarity, we have added the following explanatory sentence to the Results section: "**Using scale totals as indicators allowed us to integrate the three measures while maintaining a parsimonious measurement model.**"

In contrast, child internalizing problems were assessed using the Strengths and Difficulties Questionnaire (SDQ) internalizing subscale, which includes only six items. Given the relatively small number of items, we specified a latent factor based directly on the individual items and then derived factor scores.

Revision in manuscript:

- Page 15 (lines 356–357)

Child internalizing problems: Why is peer problems considered as internalizing problems? This is also not in line with the definition provided by authors.

Response:

In the SDQ framework, internalizing problems are conventionally operationalized as the combination of the Emotional Symptoms and Peer Problems subscales (Goodman et al., 2010). This scoring approach is widely used in SDQ research and reflects the conceptualization that internalizing difficulties include both emotional distress and social withdrawal/peer difficulties. This is also consistent with the definition provided at the beginning of the manuscript ("child internalizing problems, i.e., symptoms of anxiety, social withdrawal, and low mood").

To clarify this point, we have now added the following sentence to the manuscript: "**In the SDQ framework, internalizing problems are conventionally operationalized as the combination of these two subscales (Goodman et al., 2010).**" We have also added example items from both subscales to make the operationalization clearer: "using the 5-item Emotional Symptoms **(e.g., 'many worries, often seems worried')** and 5-item Peer Problems **(e.g., 'rather solitary, tends to play alone')** subscales of the Strengths and Difficulties Questionnaire (SDQ; Goodman, 2001)."

New reference:

- Goodman, A., Lamping, D. L., & Ploubidis, G. B. (2010). When to use broader internalizing and externalizing subscales instead of the hypothesized five subscales on the Strengths and Difficulties Questionnaire (SDQ): Data from British parents, teachers and children. *Journal of Abnormal Child Psychology*, *38*, 1179–1191. https://doi.org/10.1007/s10802-010-9434-x

Revision in manuscript:

- Page 13 (lines 301–305)

Was Measurement invariance tested across sites?

Response:

Yes. First, as mentioned above, we examined measurement invariance for each of the three survey measures used to assess parental distress (K10, PHQ-9, and GAD-7) across the English, Hong

Kong, and Mainland China samples. These analyses established partial scalar invariance for each measure. Details are provided in the Supplementary Materials and are explicitly noted in the main text (Page 13, lines 297–298). Second, for child internalizing problems, our recently published work using the same sample has established partial scalar invariance of the SDQ internalizing subscale across the three sites. This is noted in the main text (Page 14, lines 315–316). We have also added a table in the Supplementary Materials summarizing the full results of the measurement invariance analyses. Third, across-site measurement invariance cannot be tested for the observational measures of positive control (a 0 – 7 global rating) and representational mind-mindedness (a frequency count), as each is represented by a single observed indicator. This point is also explicitly stated in the main text (Page 17, lines 402–404).

Revision in manuscript:

- Page 14 (lines 315–316)
- Page 15 (line 358)
- Supplementary Materials, Page 6, Table S3

In Table 1, what is the difference between degree and social ladder? And how do participants in MC more often have a high degree but a lower average score on the social ladder?

Response:

Thank you for raising this point for clarification. In the original manuscript, "degree education (%)" referred to the percentage of parents with a bachelor's degree or higher in each site, whereas "social ladder" referred to parents' perceived standing on a 10-rung social ladder (Singh-Manoux et al., 2003). Thus, these variables capture different constructs: the former reflects objective educational attainment, while the latter reflects subjective social status. To avoid potential confusion, we have revised the terminology in our tables. Specifically, "degree education (%)" has been renamed "**parent education (% degree-level)**" and "social ladder" has been renamed "**perceived social standing**". These definitions have also been clarified in the Table 1 note.

Regarding the reviewer's question about why participants in mainland China reported relatively high levels of degree-level education but somewhat lower perceived social standing, we note that subjective social status does not necessarily correspond directly to educational attainment. Individuals may evaluate their social standing relative to broader contextual factors such as income, occupational prestige, household income, satisfaction with standard of living, and feeling of financial security regarding the future (Singh-Manoux et al., 2003). As a result, it is possible for parents with relatively high educational qualifications to report lower perceived social standing when comparing themselves to others within their local social context.

Revision in manuscript:

- Page 25, Table 1
- Page 27, Table 3
- Page 29, Table 4
- Supplementary Materials, Page 12, Table S7

The association between parental distress and parental mind-mindedness is only discussed for England, why not for the other sites? (line 289)

Response:

Thank you for raising this point. The associations between parental distress and mind-mindedness were estimated for all three sites. In the original text, we focused on the significant association observed in England while noting that the associations in Mainland China and Hong Kong were not significant. To improve clarity, we have now revised the text to explicitly report the non-significant associations in Mainland China and Hong Kong. We also clarify that a single-paper meta-analysis indicated that the strength of these associations differed significantly across the three sites.

Revision in manuscript:

- Page 17 (lines 396–399)

I would consider conducting the meta-analysis on the regression coefficients instead of the correlations. Now, much of the results is based on correlations, but the authors can also control for confounders and then conduct a meta-analysis.
Response:<br>In the current analyses, we used **partial** correlations (*pr*) as the effect size for the single-paper meta-analyses. These partial correlations are already adjusted for relevant demographic covariates (child age and gender; parent age, gender, education, and perceived social standing), and, where applicable, for parental verbosity (i.e., non-mental frequency counts in the mind-mindedness analyses). As such, they reflect covariate-controlled associations.<br>We chose to meta-analyze partial correlations because they provide standardized, directly comparable effect sizes across sites, while maintaining a parsimonious analytic framework. This approach is consistent with prior applications of single-paper meta-analysis, where standardized and covariate-adjusted indices are used to summarize within-study associations. We agree that meta-analyzing regression coefficients represents an alternative approach. However, because regression coefficients can vary in scale and interpretation depending on model specification, partial correlations offer a more comparable metric across sites in the present context. To clarify this rationale, we have now added a brief explanatory text: "**Partial correlations (*pr*) were used as standardized, covariate-adjusted effect sizes to enable tests of heterogeneity across sites.**"<br><br>Revision in manuscript:<br>▪ Page 14 (lines 333–324)
In Table 4, please put the control variables on top, next the main effects and then the moderators.
Response:<br>Thank you for this helpful suggestion. We have revised Table 4 (and Table S4 in the Supplementary Materials) to present the variables in the requested order, with the control variables listed first, followed by the main effects, and then the moderators.<br><br>Revision in manuscript:<br>▪ Page 29, Table 4 (variable order has been changed, as requested)<br>▪ Supplementary Materials, Page 12, Table S7 (variable order has also been changed, for consistency)
The authors find a significant moderator in England (distress* mind mindedness) and China (distress* control), but only visualize the simple slopes for positive control. Please only visualize the slopes for significant interactions, and also include the simple slope for distress* mind-mindedness for England.
Response:<br>Apologies for the lack of clarity in the original figure. In the revised Figure 2, we have now clearly displayed the significant interaction between parental distress and mind-mindedness in the English sample, alongside the significant interaction between parental distress and positive control in the Mainland China sample. At the same time, we have chosen to retain the full set of simple slopes across all sites and moderators. This is because including both significant and non-significant slopes provides important contextual information about the presence or absence of moderation effects across sites, thereby avoiding selective visualization based solely on statistical significance.<br><br>To improve clarity, we have revised the figure title to "**Visual representations of simple slopes illustrating two-way interactions between parental distress and (a) mind-mindedness and (b) positive control, by site**" and added the following figure note: "***Note.* Lines represent model-estimated simple slopes of parental distress predicting child internalizing problems at low (−1 *SD*), medium (*mean*), and high (+1 *SD*) levels of mind-mindedness. Panels represent levels of positive control at low (−1 *SD*), medium (*mean*), and high (+1 *SD*). Axis scales are site-specific. Each model included both interaction terms (mind-mindedness × distress; positive control × distress), allowing their effects to be estimated simultaneously. Formal simple slope**

tests were conducted only for statistically significant interaction effects; confidence intervals for all estimates are reported in Table S8."

We have also clarified in the Results section: "Figure 2 displays the predicted values of internalizing problems based on the conditional effects of parental distress **at low (1 *SD* below the *mean*), medium (within 1 *SD* of the *mean*), and high (1 *SD* above the *mean*) levels of each moderator, with the other moderator held constant**." We hope these revisions improve the interpretability of the figure.

Revision in manuscript:

- Page 18 (lines 426–427)
- Page 30, Figure 2

I wonder to what extent mind-mindedness is a cultural phenomenon that fits with the way in which Minority world parents raise their children – the high mean for English parents gives an indication that this may just not be the way in which non-minority world parents parent their children.

Response:

While this perspective makes sense, it is challenged by work that indicates a culturally universal role for mentalizing. In our original manuscript (Page 8) we tried to make this point: "As such, mind-mindedness may play a similar function across different sites, despite marked site contrasts in mean levels (Aival-Naveh et al., 2019)." In the revised manuscript, we have tried to strengthen the justification for this position by adding the following text: "**Supporting this view, our previous findings demonstrate that, across sites, within-site individual differences in mind-mindedness show similarly sized associations with individual differences in (a) positive parenting (REFERENCE MASKED A) and (b) prenatal bonding and safety from violence (REFERENCE MASKED B).**"

Revision in manuscript:

- Pages 8–9 (lines 188–191)

Reviewer 2

The article titled "Raising Resilience: Do positive parenting and mind-mindedness mitigate effects of exposure to parental distress" was a pleasure to read. The authors aimed to identify cross-context associations between positive control and parent mind mindedness on buffering the association between parental psychological distress and child internalizing problems across cultural contexts. The study is well powered, and the authors were thoughtful about measurement invariance assessments in cross-context comparisons of results. The authors also utilized multiple methods to assess study constructs. This will clearly be a great contribution to the literature on cross-cultural parenting behaviors and their contextually specific effects on child mental health. I raise the following mostly minor suggestions with the goal of strengthening the manuscripts contribution.

Response:

We thank the reviewer for this generous and encouraging assessment of our work. We appreciate the positive feedback and the reviewer's thoughtful suggestions.

Specific comments:

1) The authors should consider changing the title. The authors do not thoroughly discuss the concept of resilience in the text of the manuscript and instead appear to be discussing risk for psychopathology. If authors wish to maintain resilience framing, they should consider a more explicit discussion of resilience theory in the manuscript.

Response:

We appreciate this suggestion, which is well grounded. We have now changed the title to: "**Breaking the Cycle of Distress via Buffering Effects of Positive Control and Mind-Mindedness**." We hope that this provides a more accurate reflection of our study findings and complies with the no-punctuation guidance.
2) The manuscript would benefit from incorporating a few additional literatures into the introduction and discussion of results:<br>a. The authors very minimally discuss the role of child age in the current analyses. Please consider describing the developmental implications of studying internalizing disorders in preschool/early elementary aged children. Please also consider the specific developmental role of these specific parenting strategies at this age.
Response:<br>We agree that this was an omission (that reflected the tight page limit we were following in our original submission) and we welcome this invitation to describe the developmental implications of involving children making the transition to school. In the introduction, we have added the following text: "**Alongside expanding the geographical scope of research on mind-mindedness, our current study also helps to increase this field's developmental scope through its focus on children making the transition to school, as previous studies have focused heavily on infancy, toddlerhood and the early preschool years (Aldrich et al., 2021). Recruiting children at the transition to school also enabled us to investigate a period in which children face multiple new situations and social challenges that can elicit feelings of both anxiety and depression (Parkes et al, 2016). Here it is worth noting that meta-analytic findings on parental mentalizing and child externalizing problems (Nieto-Retuerto et al., 2025) supports the promise of this developmental focus, as links between parental mentalizing (indexed either by mind-mindedness or by the broader measure of parental reflective functioning) and child externalizing problems are stronger in middle childhood (r = -.33) than in early childhood (r = -.11) or adolescence (r = -.13). As these authors note, these contrasts may reflect age-related differences in both children's developmental competencies and in the relative salience of contextual effects.**"<br><br>New reference:<br>- Nieto-Retuerto, M., Torres-Gomez, B., & Alonso-Arbiol, I. (2025). Parental mentalization and children's externalizing problems: A systematic review and meta-analysis. *Development and Psychopathology*, *37*(4), 1804–1820. https://doi.org/10.1017/S0954579424001391<br><br>Revision in manuscript:<br>- Pages 3–4 (lines 54–67)
b. The authors importantly discuss the importance of studying the study constructs in non-western countries. It would be beneficial if the authors elaborated more on how they expect these behaviors to vary based on cultural ideals and values. What specific aspects of each context may shift parenting values/goals/behaviors? How might future studies do a better job of capturing within context variation in these cultural attributes/values/beliefs that may influence these differences in associations?
Response:<br>We collected data during the COVID-19 pandemic and were struck by site contrasts in government measures to restrict transmission (e.g., school closures were much longer in duration in Hong Kong, and families reported significantly higher levels of COVID-related stress). We referred to this in the Discussion section and noted that threshold effects (e.g., diminishing returns beyond a "good enough" level of parenting quality), coupled with differences in parents' educational aspirations, may have also contributed to site-specific findings. In the revised manuscript, we go beyond these factors to consider site differences in parents' ethnotheories, adding the text below to the Discussion section: "**Evidence for cultural contrasts in parents' cognitions also comes from a recent study of parental ethnotheories – often implicit, culturally informed belief systems –**

**that involved 108 parents of 5-year-old children living in Mainland China or the USA (Ip et al., 2024). Consistent with a 'universality without uniformity' model, parents showed both common and distinct concerns. In particular, both groups of parents viewed social insensitivity as most worrying, and both groups were much less concerned about physical weakness. At the same time, the two groups showed some differences in parental values: child aggression / disruptive behavior emerged as key foci of concern for parents in the USA, while cognitive / motivational difficulties evoked greater concern among Chinese parents. Moreover, beliefs differed in that misbehavior was typically attributed to transient internal states by American parents, but to social factors or temperament by Chinese parents. Assessing parental ethnotheories alongside mind-mindedness and direct observations of positive control may therefore offer a promising avenue for future studies to explore site contrasts in potential buffers for breaking the intergenerational cycle of distress**." <br>New reference: - Ip, K. I., Heng, J. A., Lin, J., Shi, J., Li, W., & Olson, S. (2024). Parents' ethnotheories of the nature and causes of children's misbehaviors: A comparison of mothers across two cultures. *International Journal of Behavioral Development*, *48*(5), 385–397. https://doi.org/10.1177/0165025424123997 <br>Revision in manuscript: ▪ Page 21–22 (lines 503–516)
3) Do the authors have available information about participant race/ethnicity across study sites? Although they are living in specific countries/regions they could still vary by racial/ethnic makeup or history which can also shift cultural expectations and practices in parenting.
Response: Thank you for raising this point. Information on participants' race / ethnicity was collected and is now reported for samples across all study sites. Specifically, participants in the Hong Kong and Mainland China samples were predominantly Han Chinese (> 90%). The English sample was primarily White (70%), with 9% identifying as mixed or multiple ethnic groups, 5% as Asian British, 1% as Black British, and 15% undisclosed. <br>Revision in manuscript: ▪ Page 10 (lines 229–232)
4) Please provide more information about your education and social ladder measure.
Response: Thank you for your suggestions. We have now added more detailed information about the measures of parent education and perceived social standing. For parent education, parents reported their highest level of educational attainment (1 = *junior secondary school or below*, 2 = *senior secondary school*, 3 = *associate degree*, 4 = *bachelor's degree*, 5 = *master's degree*, 6 = *PhD*). For parents' perceived social standing, completed the Ladder of Subjective Social Status (Singh-Manoux et al., 2003) by rating their perceived social standing within their local cultural context on a 10-point ladder, where the top represents those with the highest levels of educational qualification, income status, and employment and the bottom represents those with the lowest. We also recognized that the terminology "social ladder" can be potentially confusing; thus, we have renamed this variable "**perceived social standing**" in the main text and tables. <br>Revision in manuscript: ▪ Page 14 (lines 321–326)
5) On line 416 the authors describe "positive mind-mindedness" please further define or describe this construct.
Response:

We thank you for noting this ambiguity. In the revised manuscript, we have extended this sentence to make it clearer: "**positive trajectories of emotional adjustment were unrelated to overall mind-mindedness, but did appear associated with *positive* mind-mindedness, that is, the proportion of mentalistic child descriptions that were positive in valence**".

Revision in manuscript:

- Page 23 (lines 546–549)

6) In the discussion, the authors should re-emphasize the limitations in internalizing disorder measurement in some of the study contexts.

Response:

As noted in our earlier response to Reviewer 1, our recently published work using the same sample established partial scalar invariance of the SDQ internalizing subscale across the three sites. This is reported in the main text (Page 13, lines 310–311), and we have added a table in the Supplementary Materials summarizing the full measurement invariance results. In addition, we have now expanded the Discussion to more explicitly acknowledge the need for cultural adaptations for child adjustment measurement by adding the following text: "**In line with prior work (REFERENCE MASKED C), we established partial scalar invariance for the Strengths and Difficulties Questionnaire's internalizing subscale across sites, although this required the removal of four items. Similar item reductions have been reported in cross-cultural studies using the SDQ, often reflecting differences in item interpretation (e.g., reverse-worded items; Duinhof et al., 2020) or context-specific influences (e.g., prolonged school closures during the pandemic; Foley et al., 2023). These changes underscore the need for further cultural adaptation to enhance the cross-cultural applicability of measures of child adjustment.**"

New references:

- Duinhof, E., Lek, K., De Looze, M., Cosma, A., Mazur, J., Gobina, I., Wustner, A., Vollebergh, W. A. M., & Stevens, G. (2020). Revising the self-report strengths and difficulties questionnaire for cross-country comparisons of adolescent mental health problems: The SDQ-R. *Epidemiology and Psychiatric Sciences*, *29*, e35. https://doi.org/10.1017/S2045796019000246
- Foley, S., Ronchi, L., Lecce, S., Feng, X., Chan, M. H. M., & Hughes, C. (2023). Cross-cultural equivalence of parental ratings of child difficulties during the pandemic: Findings from a six-site study. *International Journal of Methods in Psychiatric Research*, *32*, e1933. https://doi.org/10.1002/mpr.1933

Revision in manuscript:

- Page 13 (lines 301–305)
- Page 14 (lines 315–316)
- Page 19 (lines 454–461)
- Supplementary Materials, Page 6, Table S3

7) Figure 2 is a bit challenging to follow. Perhaps consider relabeling to make it clear what the context of the stacked graph is. If possible, reduce use of acronyms and potentially find a way to denote what are significant differences based on the simple slopes analysis. Did the authors conduct a three-way interaction? If not, please consider showing the two-way interaction only in separate graphs. If you did, please report the three-way interaction in Table 4.

Response:

Thank you for this helpful suggestion. To clarify, our analyses did not test a three-way interaction among parental distress, mind-mindedness, and positive control. Rather, we estimated two two-way interactions (distress × mind-mindedness; distress × positive control), which were included simultaneously within the same regression model in each site. In response to the Reviewer's suggestion, we have revised Figure 2 to improve clarity in three ways. First, we replaced acronyms with full variable labels (e.g., "Mind-Mindedness," "Positive Control," "Parental Distress," "Child Internalizing Problems"). Second, we added clearer panel labels to explicitly indicate that panels represent levels of positive control and lines represent levels of mind-mindedness. Third, we

revised the figure title to "**Visual representations of simple slopes illustrating two-way interactions between parental distress and (a) mind-mindedness and (b) positive control, by site**" and added the following figure note: "***Note.*** **Lines represent model-estimated simple slopes of parental distress predicting child internalizing problems at low (−1 *SD*), medium (*mean*), and high (+1 *SD*) levels of mind-mindedness. Panels represent levels of positive control at low (−1 *SD*), medium (*mean*), and high (+1 *SD*). Axis scales are site-specific. Each model included both interaction terms (mind-mindedness × distress; positive control × distress), allowing their effects to be estimated simultaneously. Formal simple slope tests were conducted only for statistically significant interaction effects; confidence intervals for all estimates are reported in Table S8.**" In addition, we have also added explanatory sentence to the Results section: "Figure 2 displays the predicted values of internalizing problems based on the conditional effects of parental distress **at low (1 *SD* below the *mean*), medium (within 1 *SD* of the *mean*), and high (1 *SD* above the mean) levels of each moderator, with the other moderator held constant**." We hope these revisions improve the interpretability of the figure while maintaining fidelity to the analytic model.

Revision in manuscript:

- Page 18 (lines 426–427)
- Page 30, Figure 2

**Associate Editor**
Your manuscript titled "Breaking the Cycle of Distress via Buffering Effects of Positive Control and Mind-Mindedness" has now been seen by our reviewers, whose comments appear below. In light of their advice I am delighted to say that we are happy, in principle, to publish a suitably revised version in *Communications Psychology*.<br>We therefore invite you to revise your paper one last time to address the remaining concerns of our reviewers and a list of editorial requests. At the same time we ask that you edit your manuscript to comply with our format requirements and to maximize the accessibility and therefore the impact of your work.
We are delighted that the manuscript is considered suitable for publication in principle, and very grateful to you and the reviewers for the constructive and thoughtful feedback. We have carefully revised the paper to address all editorial requests and reviewer comments and believe that it now fully complies with the journal's formatting and accessibility guidelines.

**Reviewer 2**
The reviewer assessed a revision of the article now titled "Breaking the Cycle of Distress via Buffering Effects of Positive Control and Mind-Mindedness". The authors aimed to identify cross-context associations between positive control and parent mind mindedness on buffering the association between parent distress and child internalizing problems across cultural contexts. The authors were very responsive to reviewer comments and have strengthened the manuscript. I raise the following suggestions to further strengthen the author's contribution:
Below, we provide a detailed, point-by-point response outlining how each suggestion has been addressed.
Specific comments:<br>1) In the abstract the authors define positive control as autonomy support. This could use a more specific definition about what autonomy support is or manifests as in measurement/observation.
To provide a more specific and concrete definition of autonomy support, the revised text in the abstract now reads: "Promising candidates include positive control (i.e., autonomy support, **indexed by parental encouragement, praise, and open-ended questions**) and representational mind-mindedness (the propensity for parents to view children as independent agents)."<br>▪ Page 1 (line 4)
2) In the introduction on page 2 the authors discuss "parental buffers" but are not clear about what parenting is buffering. Please consider clarifying.
We now explicitly state that these parental factors are hypothesized to mitigate the risk of child internalizing problems: "Here, we consider two possible parental buffers **that may mitigate the risk of child internalizing problems**: positive control and mind-mindedness."<br>▪ Page 2 (lines 25–26)
3) At the top of page 4 the authors report "the relative salience of contextual effects" but it is unclear what they are referring to in that sentence.
To clarify what is meant by "contextual effects", the revised text reads: "As these authors note, these **age-related** contrasts may reflect differences in both children's developmental competencies and in the relative salience of contextual effects**, as environmental support is likely to matter most during periods of transition, such as starting school**."<br>▪ Page 4 (lines 68–71)
4) On page 7 the authors report that "parental depression was only associated with reduced mind-mindedness if infants show low levels of distress", and "parenting stress was associated with low

mind mindedness in the context of high social support". The authors interpret this finding as mind-mindedness as being difficult in times of stress/parent internalizing symptoms. To me it suggests that in the context of stressful circumstances (e.g., high infant distress or low social support), that parent internalizing symptoms of distress was not associated with mind-mindedness. The findings as presented do not allow us to infer anything about how circumstances of stress are related to mind-mindedness abilities. Can the authors please clarify these findings and descriptions in the text. Was there a main effect of the "stress" circumstance on mind-mindedness?
We have revised this section to clarify the absence of main effects. Specifically, the revised text reads: "However, other studies report moderating effects for both child and contextual characteristics. For example, in a longitudinal study of 50 Turkish mothers, prenatal depression **showed no overall association with interactive mind-mindedness at 12-months post-partum, but there was a significant interaction effect, such that prenatal depression** was only associated with reduced mind-mindedness **in the context of low infant negative emotionality** (Corapci et al., 2024). Likewise, in a study involving 65 Italian mothers of both full-term and premature infants, parenting stress **showed no main effect association with interactive** mind-mindedness **at 6-months postpartum, but was associated with reduced mind-mindedness** if mothers received medium or high levels of social support (Suttora et al., 2021). **That is, maternal perception of emotional support from close others promoted mind-mindedness, but only in the context of low or mild parenting stress.** Together, these findings suggest that tuning into others' thoughts and feelings may be difficult under conditions of stress, even in the absence of symptoms of depression or anxiety."<br>▪ Page 7 (lines 145–153)
a. For this paper's analyses can the authors confirm in text if there was any evaluation or main effect of COVID-19 stress exposure on level of mind-mindedness?
We did not evaluate the main effect of COVID-19 stress exposure on parental mind-mindedness in the current analyses. Instead, we examined parental distress as the key predictor of child internalizing problems and consider mind-mindedness and positive control as potential moderators.
5) Page 9 the authors refer to "basic psychological needs model". Please define or explain this model.
We have revised the text to clarify what is meant; specifically, the revised text reads: "Drawing on **the model that wellbeing hinges on the satisfaction of** basic psychological needs **for relatedness, autonomy and competence**, we posit that child internalizing problems are likely to be reduced when parents who, despite their own problems of depression or anxiety, can tune into their child's interests and feelings."<br>▪ Page 9 (lines 200–201)
6) On page 19 the authors interpret the association between parental distress and positive control as being significant across site as evidence for more within-culture variability than between culture variability. I am not sure this analysis points to that exact evidence. That sort of question may be better answered by looking at spread and variability in the data. This question may also be answered by running a multilevel model with ICC values or random-effect ANOVA for example, to evaluate if between or within country variance in parenting.
We agree that our original wording overstated what could be inferred, as demonstrating that the association between parental distress and positive control is consistent across sites does not constitute a direct test of the relative magnitude of within- versus between-culture variability. In response to the reviewer's suggestion to examine variability more closely, Table 2 indicates that variability in psychological distress is indeed greater within sites than between sites; however, this pattern is not evident for positive control. Accordingly, in the revised manuscript we describe our findings as **consistent** with prior work suggesting substantial within-culture variability, rather than as providing direct evidence for it.<br>▪ Page 20 (lines 474–477)

7) On page 20 the authors report that positive control only buffered the effects of parent distress on child internalizing symptoms in mainland China. This is interesting given that the effect sizes appear to be equivalent across sites. The authors astutely point towards average level differences in positive control across sites with Mainland China being almost 2 points lower on average on this assessment. I appreciate the "good enough" positive control/parenting interpretation, but given the cross-cultural evaluation goals of this study, I wonder if the authors have considered any cultural differences in Mainland China that may play a role in these findings.
Thank you for this thoughtful comment. We have expanded this section to more explicitly consider culturally grounded interpretations. The revised text reads: "As the magnitude of this moderation effect showed no significant site contrasts that might suggest culturally-specific influences, the null results in England and Hong Kong (both characterized by higher mean levels of positive control) may indicate that variability in positive control has limited influence on the association between parental distress and child internalizing problems, beyond a threshold 'good enough' level. **Another plausible interpretation hinges on how positive control operates within the family context across cultures. Meta-analytic evidence synthesizing findings from 108 independent data sets across 22 cultures shows that collectivism strengthens the internalization of socially mediated experiences, amplifying their impact on psychological outcomes (e.g., the internalization of stigma; Ben et al., 2021). By analogy, positive control in Mainland China may be more salient to children's meaning-making processes, thereby more effectively buffering the impact of parental distress.**"<br>▪ Page 21 (lines 499–500, 502–510)<br><br>New reference:<br>- Ben, C. L., Chio, F. H., Mak, W. W., Corrigan, P. W., & Chan, K. K. (2021). Internalization process of stigma of people with mental illness across cultures: A meta-analytic structural equation modeling approach. *Clinical Psychology Review*, *87*, 102029. https://doi.org/10.1016/j.cpr.2021.102029
8) On page 21 the authors describe different parenting concerns of priorities for children's behaviors. Interestingly, most of these examples would fall under concerns for externalizing behaviors. I wonder if there are any studies about parenting values for internalizing sorts of behaviors?
We have expanded this section to include research on shyness, a construct closely linked to internalizing problems, to illustrate how parenting values and cultural norms may differentially shape the interpretation and implications of such behavior across contexts. Specifically, the revised text reads: "**Of relevance to our study's focus on internalizing problems, cross-cultural contrasts have also been noted in relation to shyness in children. For example, in a study of families with 8- to 10-year-old children living in Canada (*n* = 149) or China (*n* = 145), Kong et al. (2026) distinguished between two forms of shyness (anxious and regulated) and showed that while anxious shyness showed a similar relationship with both depression and aggression in both sites, regulated shyness was only related to (low levels of) depression and aggression in Canadian children. This suggests that dispositional factors are differentially salient for Canadian children, whereas for Chinese children, regulated shyness is typically a response to external influences that promote cultural norms of modesty and harmony (Xu et al., 2020).**"<br>▪ Pages 22–23 (lines 533–542)<br><br>New references:<br>- Kong, X., Brook, C. A., Zhou, T., Liu, J., & Schmidt, L. A. (2026). A cross-cultural study of different types of shyness: Associations with depression and aggression in Canadian and Chinese children. *Social Development*, *35*(2), e70052. https://doi.org/10.1111/sode.70052<br>- Xu, Y., Stacy, T., Krieg, A. (2020). The many faces of shyness in childhood across cultural contexts. In L. A., Schmidt & K. L. Poole (Eds.), *Adaptive shyness: Multiple perspectives on behavior and development* (pp. 219–237). Springer, Cham. https://doi.org/10.1007/978-3-030-38877-5_12

9) When discussing mind-mindedness findings in the discussion, the authors do not provide an explanation about site-based differences that may lead to differences in association. Please consider adding.
We agree that it is important to consider potential site-based differences that may underlie variation in the observed associations. In fact, we had included an initial interpretation in the Discussion (Page 23, lines 552–558): "Notably, the moderation effect for mind-mindedness was specific to England, perhaps reflecting across-site contrasts in the associations between parental distress and mind-mindedness. Given that a proclivity to focus on mental states may reduce the likelihood of parents viewing child behaviors as irritating or difficult (McMahon & Bernier, 2017), it may be that the strong emphasis on restraint of emotional expression within Confucian societies (including Hong Kong and Mainland China; Chan, 2020) makes mind-mindedness superfluous in terms of limiting children's exposure to parental negative affect."
10) For all variables, were there main effects of mind-mindedness, positive control, and parent psychological distress on child internalizing outcomes? Did you assess these models WITHOUT interactions and then assess if model fit improved with the inclusion of moderation effects?
Thank you for this question. All models were specified as just-identified (i.e., saturated), meaning that a single set of parameter estimates perfectly fits the observed data (Brown, 2015). As a result, overall model fit indices are not informative, and model fit cannot be compared across models with and without interaction terms. To address the reviewer's concern, we have now included the baseline main-effect models (i.e., without interaction terms) in the Supplementary Materials (Table S7) and have clarified this in the manuscript.<br>▪ Page 18 (lines 421–422)<br>▪ Supplemnatry Materials (Page 11, Table S7)
11) One limitation of the study may be that all samples were made up primarily by the majority race/ethnicity in their country. This may include an expanded discussion around how within country culture can vary and may lead to heterogeneity within associations both within and across countries.
We agree that our samples, which were relatively homogeneous in terms of ethnicity within each site, may not capture meaningful within-country cultural variability. We have expanded the limitations section: "**Fifth, our study samples were ethnically relatively homogeneous within each site; future work is needed to integrate within- and between-site cultural contrasts in parenting.**"<br>▪ Pages 24–25 (lines 590–592)
