# Supplementary material for: Cross-cultural evidence for positive control and mind-mindedness as buffers for parental distress
Source: Commun Psychol. 2026 Jun 17;4:133. doi: 10.1038/s44271-026-00484-x (PMC13634921; doi:10.1038/s44271-026-00484-x)
Supplement: Supplementary file 2 [file 44271_2026_484_MOESM2_ESM.pdf]

Supplementary Materials for

# Cross-Cultural Evidence for Positive Control and Mind-Mindedness as Buffers for Parental Distress

This file includes the following materials:


Partial scalar invariance across sites for parental distress measures

Table S1. *Tests of internal consistency and measurement invariance for parental distress measures across sites*

Table S2. *Selection of SDQ items for a one-factor measurement model of internalizing problems*

Table S3. *Tests of measurement invariance for the 6-item internalizing subscale of the SDQ across sites*

Table S4. *Standardized factor loadings for the latent parental distress construct*

Table S5. *Standardized factor loadings for the latent child internalizing problems construct*

Table S6. *Bivariate correlations with confidence intervals*

Sensitivity analyses using Bayesian estimation

Table S7. *Robust maximum likelihood estimates for the baseline main-effect models predicting children's internalizing problems*

Table S8. *Bayesian estimates for the moderation models*

Table S9. *Robust maximum likelihood estimates for the simple slope analyses*

Figure S1. *Residuals vs. fitted plots testing linearity of associations between parental distress and mind-mindedness*

Figure S2. *Residuals vs. fitted plots testing linearity of associations between parental distress and positive control*

Figure S3. *Forest plot displaying consistent effect sizes for the link between parental distress and positive control across sites*

Figure S4. *Forest plot displaying varying effect sizes for the link between parental distress and mind-mindedness across sites*

**Partial scalar invariance across sites for parental distress measures**

We used multi-group confirmatory factor analysis (CFA) in M*plus* Version 8 (Muthén & Muthén, 2017) to test cross-site measurement invariance for each of the three parental psychological distress measures. We applied a robust maximum likelihood estimator in each model to account for non-normal distributions of our indicators. Model fit was evaluated using four criteria (Brown, 2015): Root Mean Square Error of Approximation (RMSEA) < 0.08, Comparative Fit Index (CFI) > 0.90, Tucker–Lewis Index (TLI) > 0.90, and Standardized Root Mean Residual (SRMR) < 0.10. In addition, model parameters were considered invariant if nested model comparisons showed a non-significant change of < 0.015 in RMSEA, and < 0.01 in CFI (Chen, 2007); equality constraints across sites were considered acceptable if the change in SRMR was < 0.03 for metric invariance or < 0.015 for scalar invariance (Chen, 2007).

***Kessler Psychological Distress Scale (K10)***

In line with previous research (Lace et al., 2019), we specified a two-factor model, with five indicators (items 5, 6, 7, 8, 9) loading onto the depression factor and five indicators (items 1, 2, 3, 4, 10) loading onto the anxiety factor, allowing the two factors to correlate. The configural invariant model, in which the same factor structure was simultaneously estimated across sites while allowing parameters to differ, provided an acceptable fit to the data, RMSEA = 0.081, CFI = 0.957, TLI = 0.935, SRMR = 0.044. The subsequent metric invariant model, which constrained factor loadings to be equal across sites, also provided a good fit to the data, RMSEA = 0.080, CFI = 0.951, TLI = 0.937, SRMR = 0.064. Given the non-significant change in model fit relative to the less parsimonious model, it can be inferred that the questionnaire measure of nonspecific psychological distress have the same structure and meaning for parents from different cultural groups (Brown, 2015). In the scalar invariant model, however, imposing additional constraints of equal intercepts resulted in a significant decrease in model fit, ΔRMSEA = 0.018, ΔCFI = 0.037. As full measurement invariance in all steps is often not supported, it is common to proceed with tests of mean differences or relations between constructs using a partially invariant solution (Putnick & Bornstein, 2016). Partial (metric or scalar) invariance modeling allows estimates for a subset of non-invariant parameters (loadings or intercepts) to vary across groups while constraining invariant parameters to equality (Brown, 2015; Putnick & Bornstein, 2016). In accordance with modification indices, we allowed three indicator intercepts (items 3, 8, 9) to be freely estimated, achieving partial scalar invariance with the proposed criteria for ΔRMSEA, ΔCFI, and ΔSRMR met. In other words, the two-factor K10 model exhibited partial scalar invariance, with equal form, equal loadings, and partially equal intercepts across the English, Hong Kong, and mainland Chinese sub-samples.

***Patient Health Questionnaire (PHQ-9)***

We first specified a one-factor measurement model, with all nine indicators loading onto the same factor. The configural invariant model provided an acceptable fit to the data, RMSEA = 0.074, CFI = 0.934, TLI = 0.912, SRMR = 0.048. The subsequent metric invariant model also provided a good fit to the data, RMSEA = 0.067, CFI = 0.935, TLI = 0.928, SRMR = 0.071, with the non-significant change in model fit compared to the less parsimonious model. In the scalar invariant model, however, imposing additional constraints of equal intercepts across groups resulted in a significant decrease in model fit, ΔRMSEA = 0.013, ΔCFI = 0.042. In accordance with modification indices, we allowed three indicator

intercepts (items 1, 5, 8) to be freely estimated in the English sub-sample and two indicator intercepts (items 1, 4) to be freely estimated in the mainland Chinese sub-sample, achieving partial scalar invariance with the proposed criteria for ΔRMSEA, ΔCFI, and ΔSRMR met. In sum, the one-factor PHQ-9 model exhibited partial scalar invariance, with equal form, equal loadings, and partially equal intercepts across all three sites.

### *Generalized Anxiety Disorder Scale (GAD-7)*

We first specified a one-factor model, with all seven indicators loading onto the same factor. The configural invariant model provided a good fit to the data, RMSEA = 0.064, CFI = 0.978, TLI = 0.967, SRMR = 0.031. The subsequent metric invariant model resulted in a significant decrease in model fit, ΔRMSEA = 0.006, ΔCFI = 0.012. In accordance with modification indices, we allowed two indicator loadings (items 2, 4) to be freely estimated, achieving partial metric invariance with the proposed criteria for ΔRMSEA, ΔCFI, and ΔSRMR met. The partial scalar invariant model also provided an acceptable fit to the data, RMSEA = 0.070, CFI = 0.961, TLI = 0.961, SRMR = 0.062. In sum, the one-factor GAD-7 model exhibited partial scalar invariance, with equal form, partially equal loadings, and partially equal intercepts across all three sites.

### *References*


Chen, F. F. (2007). Sensitivity of goodness of fit indexes to lack of measurement invariance. *Structural Equation Modeling: A Multidisciplinary Journal*, *14*, 464–504. https://doi.org/10.1080/10705510701301834

Lace, J. W., Greif, T. R., McGrath, A., Grant, A. F., Merz, Z. C., Teague, C. L., & Handal, P. J. (2019). Investigating the factor structure of the K10 and identifying cutoff scores denoting nonspecific psychological distress and need for treatment. *Mental Health & Prevention*, *13*, 100–106. https://doi.org/10.1016/j.mhp.2019.01.008

Putnick, D. L., & Bornstein, M. H. (2016). Measurement invariance conventions and reporting: The state of the art and future directions for psychological research. *Developmental Review*, *41*, 71–90. https://doi.org/10.1016/j.dr.2016.06.004

**Table S1.** *Tests of internal consistency and measurement invariance for parental distress measures across sites*

| | $\chi^2$ | *df* | RMSEA [90% CI] | CFI | TLI | SRMR | Model comparison | ΔCFI | ΔRMSEA | ΔSRMR |
|---|---|---|---|---|---|---|---|---|---|---|
| ***K10: measurement invariance*** | | | | | | | | | | |
| Cronbach's values: $\alpha_{ENG} = 0.89$, $\alpha_{HK} = 0.91$, $\alpha_{MC} = 0.93$ | | | | | | | | | | |
| M1: configural invariance | 254.51*** | 90 | 0.081 [0.069, 0.093] | 0.957 | 0.935 | 0.044 | | | | |
| M2: metric invariance | 294.15*** | 106 | 0.080 [0.069, 0.091] | 0.951 | 0.937 | 0.064 | M2 – M1 | 0.006 | 0.001 | 0.020 |
| M3: partial scalar invariance | 337.36*** | 116 | 0.083 [0.073, 0.093] | 0.942 | 0.932 | 0.065 | M3 – M2 | 0.009 | 0.003 | 0.001 |
| ***PHQ-9: measurement invariance*** | | | | | | | | | | |
| Cronbach's values: $\alpha_{ENG} = 0.88$, $\alpha_{HK} = 0.81$, $\alpha_{MC} = 0.89$ | | | | | | | | | | |
| M1: configural invariance | 205.50*** | 81 | 0.074 [0.062, 0.087] | 0.934 | 0.912 | 0.048 | | | | |
| M2: metric invariance | 218.81*** | 97 | 0.067 [0.055, 0.079] | 0.935 | 0.928 | 0.071 | M2 – M1 | 0.001 | 0.007 | 0.023 |
| M3: partial scalar invariance | 240.78*** | 108 | 0.067 [0.055, 0.078] | 0.930 | 0.930 | 0.072 | M3 – M2 | 0.005 | 0.000 | 0.001 |
| ***GAD-7: measurement invariance*** | | | | | | | | | | |
| Cronbach's values: $\alpha_{ENG} = 0.88$, $\alpha_{HK} = 0.90$, $\alpha_{MC} = 0.92$ | | | | | | | | | | |
| M1: configural invariance | 89.01*** | 42 | 0.064 [0.045, 0.082] | 0.978 | 0.967 | 0.031 | | | | |
| M2: partial metric invariance | 117.95*** | 52 | 0.068 [0.051, 0.084] | 0.969 | 0.963 | 0.061 | M2 – M1 | 0.009 | 0.004 | 0.030 |
| M3: partial scalar invariance | 145.54*** | 62 | 0.070 [0.055, 0.085] | 0.961 | 0.961 | 0.062 | M3 – M2 | 0.008 | 0.002 | 0.001 |

*Note.* RMSEA, Root Mean Square Error of Approximation; 90% CI, 90% confidence interval for RMSEA; CFI, Comparative Fit Index; TLI, Tucker–Lewis Index; SRMR, Standardized Root Mean Square Residual. ΔCFI = difference in the robust CFI; ΔRMSEA = difference in the robust RMSEA; ΔSRMR = difference in the robust SRMR. $^{***}p < .001$. $^{**}p < .01$. $^{*}p < .05$.

**Table S2.** *Selection of SDQ items for a one-factor measurement model of internalizing problems*

| | Inclusion | Exclusion |
|---|---|---|
| ***Emotion symptoms*** | | |
| Item 3: complaints of headaches | | √ |
| Item 8: many worries | √ | |
| Item 13: often unhappy and tearful | √ | |
| Item 16: nervous and clingy | √ | |
| Item 24: many fears and easily scared | √ | |
| ***Peer problems*** | | |
| Item 6: solitary play | √ | |
| Item 11: at least one good friend (rev) | | √ |
| Item 14: liked by other children (rev) | | √ |
| Item 19: picked on and bullied | √ | |
| Item 23: gets on better with adults | | √ |

*Note*. (rev) denotes items that are reverse scored.

**Table S3.** *Tests of measurement invariance for the 6-item internalizing subscale of the SDQ across sites*

| | $\chi^2$ | *df* | RMSEA [90% CI] | CFI | TLI | SRMR | Model comparison | ΔCFI | ΔRMSEA | ΔSRMR |
|---|---|---|---|---|---|---|---|---|---|---|
| ***Measurement CFA*** | | | | | | | | | | |
| M1: whole sample | 12.29 ($p$ = .139) | 8 | 0.025 [0.000, 0.051] | 0.989 | 0.979 | 0.023 | | | | |
| M2: England | 15.85* | 8 | 0.062 [0.009, 0.106] | 0.953 | 0.912 | 0.042 | | | | |
| M3: Hong Kong | 15.32 ($p$ = .053) | 8 | 0.059 [0.000, 0.103] | 0.951 | 0.909 | 0.046 | | | | |
| M4: Mainlandd China | 6.064 ($p$ = .640) | 8 | 0.000 [0.000, 0.054] | 1.000 | 1.000 | 0.028 | | | | |
| ***Multiple-groups CFA*** | | | | | | | | | | |
| M5: configural invariance | 35.75 ($p$ = .058) | 24 | 0.042 [0.000, 0.069] | 0.972 | 0.947 | 0.038 | | | | |
| M6: metric invariance | 47.27 ($p$ = .065) | 34 | 0.037 [0.000, 0.061] | 0.968 | 0.958 | 0.052 | M6 – M5 | 0.004 | 0.005 | 0.014 |
| M7: partial scalar invariance | 53.06 ($p$ = .066) | 39 | 0.036 [0.000, 0.058] | 0.966 | 0.961 | 0.053 | M7 – M6 | 0.002 | 0.001 | 0.001 |

*Note.* RMSEA, Root Mean Square Error of Approximation; 90% CI, 90% confidence interval for RMSEA; CFI, Comparative Fit Index; TLI, Tucker–Lewis Index; SRMR, Standardized Root Mean Square Residual. ΔCFI = difference in the robust CFI; ΔRMSEA = difference in the robust RMSEA; ΔSRMR = difference in the robust SRMR. $^{***}p < .001$. $^{**}p < .01$. $^{*}p < .05$.

**Table S4.** *Standardized factor loadings for the latent parental distress construct*

| | ENG | HK | MC |
|---|---|---|---|
| ***Psychological distress*** | | | |
| K10[a] | .91*** | .83*** | .94*** |
| PHQ-9[a] | .93*** | .85*** | .86*** |
| GAD-7[a] | .85*** | .89*** | .88*** |

*Note.* [a] Values represent total scores of each measure. Estimated using a robust maximum likelihood estimator. ENG = England; HK = Hong Kong; MC = Mainland China. ***$p < .001$. **$p < .01$. *$p < .05$.

**Table S5.** *Standardized factor loadings for the latent child internalizing problems construct*

| | ENG | HK | MC |
|---|---|---|---|
| ***Child internalizing problems*** | | | |
| Item 8: many worries | .85*** | .71*** | .61*** |
| Item 13: often unhappy and tearful | .59*** | .48*** | .43*** |
| Item 16: nervous and clingy | .44*** | .36*** | .30** |
| Item 24: many fears and easily scared | .50*** | .55*** | .62*** |
| Item 6: solitary play | .37*** | .36** | .33*** |
| Item 19: picked on and bullied | .22* | .30** | .32*** |

*Note.* Estimated using a robust maximum likelihood estimator. ENG = England; HK = Hong Kong; MC = Mainland China. ***$p < .001$. **$p < .01$. *$p < .05$.

**Table S6.** *Bivariate correlations with confidence intervals*

A. England

| | 1 | 2 | 3 | 4 | 5 | 6 | 7 | 8 | 9 | 10 |
|---|---|---|---|---|---|---|---|---|---|---|
| 1. Mind-mindedness[a] | – | | | | | | | | | |
| 2. Positive control | $.25^{***}$<br>[.117, 364] | – | | | | | | | | |
| 3. Psychological distress[b] | $-.16^{*}$<br>[-.277, -.037] | $-.19^{**}$<br>[-.315, -.060] | – | | | | | | | |
| 4. Internalizing problems[c] | .00<br>[-.118, .125] | -.09<br>[-.218, .042] | $.25^{***}$<br>[.125, .357] | – | | | | | | |
| *Covariates* | | | | | | | | | | |
| 5. Child age | $.11^{+}$<br>[-.010, .233] | .04<br>[-.090, .174] | .07<br>[-.051, .197] | $.16^{*}$<br>[.035, .275] | – | | | | | |
| 6. Child gender | -.06<br>[-.181, .062] | .01<br>[-.123, .139] | -.02<br>[-.140, .107] | .07<br>[-.053, .190] | $.11^{+}$<br>[-.011, .232] | – | | | | |
| 7. Parent age | .02<br>[-.106, .139] | .08<br>[-.052, .209] | -.06<br>[-.178, .070] | -.02<br>[-.145, .101] | .03<br>[-.096, .151] | -.08<br>[-.196, .047] | – | | | |
| 8. Parent gender | .01<br>[-.116, .128] | -.01<br>[-.140, .122] | -.03<br>[-.149, .098] | .08<br>[-.046, .196] | .04<br>[-.084, .162] | -.01<br>[-.127, .117] | $-.11^{+}$<br>[-.226, .017] | – | | |
| 9. Parent education | $.11^{+}$<br>[-.008, .233] | .02<br>[-.107, .154] | -.10<br>[-.220, .024] | .05<br>[-.076, .167] | .00<br>[-.128, .119] | -.02<br>[-.143, .101] | $.23^{***}$<br>[.106, .339] | -.08<br>[-.203, .040] | – | |
| 10. Perceived social standing | .08<br>[-.043, .203] | .02<br>[-.115, .150] | $-.25^{***}$<br>[-.364, -.130] | -.08<br>[-.198, .048] | -.04<br>[-.164, .085] | .04<br>[-.087, .160] | $.21^{**}$<br>[.082, .321] | -.09<br>[-.208, .037] | $.35^{***}$<br>[.237, .455] | – |
| 11. Non-mental frequency | $.40^{***}$<br>[.289, .495] | .07<br>[-.066, .195] | $-.11^{+}$<br>[-.230, .014] | -.09<br>[-.214, .028] | -.10<br>[-.218, .026] | -.01<br>[-.132, .112] | .05<br>[-.073, .172] | .08<br>[-.047, .196] | $.11^{+}$<br>[-.096, .151] | .03<br>[-.096, .151] |

B. Hong Kong

| | 1 | 2 | 3 | 4 | 5 | 6 | 7 | 8 | 9 | 10 |
|---|---|---|---|---|---|---|---|---|---|---|
| 1. Mind-mindedness[a] | – | | | | | | | | | |
| 2. Positive control | .18$^{**}$ [.054, .299] | – | | | | | | | | |
| 3. Psychological distress[b] | .11$^{+}$ [-.017, .223] | -.02 [-.151, .104] | – | | | | | | | |
| 4. Internalizing problems[c] | .03 [-.094, .147] | .05 [-.074, .178] | .25$^{***}$ [.132, .360] | – | | | | | | |
| *Covariates* | | | | | | | | | | |
| 5. Child age | .03 [-.088, .153] | -.20$^{**}$ [-.321, -.078] | -.03 [-.154, .091] | -.15$^{*}$ [-.262, -.025] | – | | | | | |
| 6. Child gender | -.09 [-.211, .028] | -.07 [-.192, .060] | .05 [-.071, .172] | .14$^{*}$ [.020, .256] | .06 [-.066, .175] | – | | | | |
| 7. Parent age | .19$^{**}$ [.069, .302] | .08 [-.044, .207] | .08 [-.045, .197] | .00 [-.124, .117] | .02 [-.106, .137] | -.06 [-.175, .065] | – | | | |
| 8. Parent gender | .16$^{**}$ [.042, .278] | .03 [-.094, .159] | .10$^{+}$ [-.019, .223] | -.08 [-.202, .038] | .00 [-.118, .125] | .04 [-.080, .161] | -.07 [-.187, .054] | – | | |
| 9. Parent education | .19$^{**}$ [.069, .302] | .28$^{***}$ [.160, .392] | -.10 [-.217, .024] | -.01 [-.129, .112] | -.08 [-.200, .041] | -.09 [-.210, .029] | .03 [-.092, .149] | -.09 [-.204, .036] | – | |
| 10. Perceived social standing | .03 [-.095, .150] | .08 [-.052, .203] | -.34$^{***}$ [-.447, -.230] | -.01 [-.134, .111] | -.09 [-.207, .038] | .00 [-.122, .124] | -.11$^{+}$ [-.226, .016] | .09 [-.039, .206] | .31$^{***}$ [.191, .414] | – |
| 11. Non-mental frequency | .27$^{***}$ [.151, .375] | .00 [-.122, .130] | -.08 [-.197, .045] | -.06 [-.176, .063] | .02 [-.099, .143] | -.03 [-.147, .094] | .02 [-.097, .144] | .07 [-.052, .189] | .09 [-.027, .212] | .05 [-.070, .175] |

C. Mainland China

| | 1 | 2 | 3 | 4 | 5 | 6 | 7 | 8 | 9 | 10 |
|---|---|---|---|---|---|---|---|---|---|---|
| 1. Mind-mindedness[a] | – | | | | | | | | | |
| 2. Positive control | .10$^{+}$ [-.006, .210] | – | | | | | | | | |
| 3. Psychological distress[b] | -.08 [-.183, .033] | -.16$^{**}$ [-.265, -.053] | – | | | | | | | |
| 4. Internalizing problems[c] | .04 [-.067, .151] | -.12* [-.221, -.006] | .26$^{***}$ [.158, .361] | – | | | | | | |
| *Covariates* | | | | | | | | | | |
| 5. Child age | .09 [-.025, .192] | -.08 [-.191, .026] | -.21$^{***}$ [-.311, -.103] | -.09 [-.198, .018] | – | | | | | |
| 6. Child gender | .07 [-.041, .176] | .10$^{+}$ [-.007, .209] | .04 [-.065, .153] | -.05 [-.158, .060] | .00 [-.107, .111] | – | | | | |
| 7. Parent age | .04 [-.069, .149] | .11$^{+}$ [-.003, .214] | -.08 [-.185, .033] | -.11$^{*}$ [-.217, .000] | .14$^{*}$ [.034, .249] | .03 [-.079, .140] | – | | | |
| 8. Parent gender | -.02 [-.130, .088] | .00 [-.108, .110] | -.02 [-.130, .088] | -.07 [-.177, .040] | -.02 [-.125, .093] | -.01 [-.119, .099] | -.04 [-.150, .068] | – | | |
| 9. Parent education | .16$^{**}$ [.047, .260] | .22$^{***}$ [.117, .324] | -.18$^{**}$ [-.281, -.070] | -.13$^{*}$ [-.236, -.022] | -.01 [-.120, .098] | .09 [-.019, .197] | .18$^{**}$ [.071, .283] | -.08 [-.184, .033] | – | |
| 10. Perceived social standing | .10$^{+}$ [-.007, .209] | .10$^{+}$ [-.007, .209] | -.15$^{**}$ [-.253, -.040] | -.08 [-.187, .029] | .02 [-.090, .128] | -.07 [-.178, .039] | -.03 [-.138, .080] | -.04 [-.147, .070] | .25$^{***}$ [.141, .346] | – |
| 11. Non-mental frequency | .59$^{***}$ [.513, .656] | .05 [-.060, .158] | -.04 [-.150, .068] | .06 [-.054, .164] | -.08 [-.192, .025] | -.09 [-.201, .015] | .06 [-.049, .169] | .05 [-.063, .155] | .05 [-.058, .159] | .03 [-.081, .137] |

*Note.* [a] Frequency of mental attributes. [b] Latent factor scores for psychological distress. [c] Latent factor scores for internalizing problems. Child and parent gender were dummy coded with male being the reference category; 95% confidence intervals are presented in brackets. $^{***}p < .001$. $^{**}p < .01$. $^{*}p < .05$. $^{+}p < .10$.

**Table S7.** *Robust maximum likelihood estimates for the baseline main-effect models predicting children's internalizing problems*

| | ENG | | | | HK | | | | MC | | | |
|---|---|---|---|---|---|---|---|---|---|---|---|---|
| | *Est.* | *SE* | *Std.Est.* | 95% CI | *Est.* | *SE* | *Std.Est.* | 95% CI | *Est.* | *SE* | *Std.Est.* | 95% CI |
| Child age | .065 | .093 | .053 | [-.095, .200] | **-.104** | .047 | -.145* | [-.268, -.021] | -.014 | .018 | -.039 | [-.137, .059] |
| Child gender | .091 | .058 | .105 | [-.026, .235] | **.078** | .035 | .152* | [.021, .283] | -.023 | .024 | -.052 | [-.153, .050] |
| Parent age | .000 | .006 | -.005 | [-.129, .119] | -.002 | .004 | -.025 | [-.143, .093] | -.004 | .004 | -.074 | [-.198, .050] |
| Parent gender | .150 | .109 | .077 | [-.033, .188] | -.101 | .061 | -.121 | [-.266, .023] | -.080 | .065 | -.077 | [-.200, .046] |
| Parent education | .041 | .030 | .086 | [-.040, .212] | -.005 | .013 | -.023 | [-.143, .096] | -.021 | .016 | -.073 | [-.180, .034] |
| Perceived social standing | -.017 | .023 | -.057 | [-.203, .089] | .006 | .011 | .034 | [-.100, .168] | -.006 | .010 | -.039 | [-.163, .085] |
| Non-mental frequency | -.005 | .003 | -.127 | [-.266, .011] | .000 | .002 | -.009 | [-.137, .119] | .001 | .003 | .034 | [-.099, .167] |
| Psychological distress | **.017** | .007 | .190* | [.037, .344] | **.013** | .004 | .264** | [.118, .411] | **.008** | .002 | .230** | [.114, .347] |
| Mind-mindedness | .006 | .005 | .110 | [-.071, .292] | .003 | .004 | .069 | [-.100, .237] | .004 | .003 | .074 | [-.045, .194] |
| Positive control | -.028 | .022 | -.085 | [-.214, .045] | .007 | .013 | .033 | [-.090, .156] | -.013 | .011 | -.066 | [-.170, .039] |

*Note. Est.* = unstandardized estimate; significant estimates are shown in bold. *Std.Est.* = standardized estimate; 95% CI = 95% confidence intervals for standardized estimates. Child and parent gender were dummy coded with male being the reference category. ENG = England; HK = Hong Kong; MC = Mainland China. $^{**}p < .01$. $^{*}p < .05$.

## Sensitivity analyses using Bayesian estimation

As a robustness check, we re-estimated the site-specific moderation models using Bayesian estimation implemented via Markov chain Monte Carlo (MCMC) sampling in M*plus* (Brown, 2015; Muthén & Muthén, 2017). Model fit was evaluated using two criteria (Brown, 2015; Muthén & Asparouhov, 2012): a posterior predictive *p* (PPP) value close to .50, and a 95% confidence interval (CI) with the lower band negative and bounds roughly symmetric around a zero fit-statistic difference (observed – replicated). Statistical significance is reflected by 95% credibility interval (CrI) that does not cover zero (Brown, 2015; Muthén & Asparouhov, 2012). Table S8 presents the full results.

In the English sub-sample, model fit was adequate, PPP = .470, 95% CI [-15.697, 15.590], which accounted for 19% of the variance in child internalizing problems. Consistent with the moderation analyses using MLR, mind-mindedness significantly attenuated the intergenerational association between psychological distress and internalizing problems, 95% CrI [-.389, -.102], whereas the moderation role of positive control was not supported, 95% CrI [-.212, .072].

In the Hong Kong sub-sample, model fit was adequate, PPP = .462, 95% CI [-13.706, 15.513], which accounted for 17% of the variance in child internalizing problems. Consistent with the moderation analyses using MLR, the intergenerational association between psychological distress and internalizing problems was not moderated by either mind-mindedness, [-.167, .084], or positive control, [-.231, .019].

In the Mainland Chinese subsample, model fit was adequate, PPP = .476, 95% CI [-14.171, 14.852], which accounted for 14% of the variance in child internalizing problems. Consistent with the moderation analyses using MLR, the moderating role of mind-mindedness was not supported, 95% CrI [-.046, .171]. The moderating role of positive control was small and borderline negative, 95% CrI [-.215, .000], indicating that higher positive control may buffer the intergenerational association between psychological distress and child internalizing problems.

### *Reference*

Muthén, B., & Asparouhov, T. (2012). Bayesian structural equation modeling: A more flexible representation of substantive theory. *Psychological Methods*, *17*(3), 313–335. https://doi.org/10.1037/a0026802

**Table S8.** *Bayesian estimates for the moderation models*

| | ENG | | | | HK | | | | MC | | | |
|---|---|---|---|---|---|---|---|---|---|---|---|---|
| | *Est.* | *SD* | 95% CrI | *Std.Est.* | *Est.* | *SD* | 95% CrI | *Std.Est.* | *Est.* | *SD* | 95% CrI | *Std.Est.* |
| Child age | .098 | .086 | [-.052, .200] | .075 | -.104 | .047 | [-.253, -.016] | -.137* | -.015 | .020 | [-.151, .065] | -.041 |
| Child gender | .086 | .058 | [-.030, .213] | .093 | .081 | .034 | [.028, .264] | .149* | -.023 | .025 | [-.151, .056] | -.050 |
| Parent age | -.001 | .007 | [-.135, .119] | -.009 | -.001 | .004 | [-.141, .104] | -.016 | -.004 | .003 | [-.170, .038] | -.066 |
| Parent gender | .143 | .131 | [-.053, .191] | .069 | -.107 | .056 | [-.242, .004] | -.122 | -.077 | .056 | [-.171, .030] | -.072 |
| Parent education | .040 | .034 | [-.056, .211] | .079 | -.008 | .016 | [-.171, .098] | -.037 | -.023 | .017 | [-.187, .038] | -.078 |
| Perceived social standing | -.017 | .022 | [-.183, .091] | -.054 | .006 | .012 | [-.097, .179] | .038 | -.006 | .009 | [-.143, .071] | -.040 |
| Non-mental frequency | -.004 | .003 | [-.221, .045] | -.094 | -.001 | .002 | [-.137, .106] | -.020 | .001 | .003 | [-.113, .145] | .012 |
| Psychological distress | .006 | .007 | [-.089, .200] | .061 | .012 | .003 | [.107, .365] | .243* | .007 | .002 | [.085, .304] | .198* |
| Mind-mindedness | .001 | .005 | [-.122, .171] | .024 | .003 | .003 | [-.053, .206] | .078 | .004 | .003 | [-.044, .217] | .088 |
| Positive control | -.022 | .023 | [-.192, .066] | -.064 | .007 | .014 | [-.098, .157] | .032 | -.015 | .012 | [-.179, .033] | -.073 |
| Mind-mindedness × Psychological distress | -.003 | .001 | [-.389, -.102] | -.247* | .000 | .000 | [-.167, .084] | -.043 | .000 | .000 | [-.046, .171] | .063 |
| Positive control × Psychological distress | -.004 | .004 | [-.212, .072] | -.072 | -.004 | .003 | [-.231, .019] | -.108 | -.004 | .002 | [-.215, .000] | -.107⁺ |

*Note*. *Est.* = unstandardized posterior point estimate; *SD* = posterior standard deviation; 95% CrI = 95% credibility interval; *Std.Est.* = standardized estimate. Child and parent gender were dummy coded (0 = male, 1 = female). ENG = England; HK = Hong Kong; MC = Mainland China. *95% CrI excludes 0. ⁺95% CrI narrowly overlaps 0.

**Table S9.** *Robust maximum likelihood estimates for the simple slope analyses*

| MM | PosControl | ENG | | | | | MC | | | | |
|---|---|---|---|---|---|---|---|---|---|---|---|
| | | *Est.* | *SE* | *t* | 95% CI | *p* | *Est.* | *SE* | *t* | 95% CI | *p* |
| Low | Low | **.035** | .008 | 4.351 | [.019, .051] | < .001 | **.009** | .003 | 3.356 | [.004, .015] | .001 |
| | Medium | **.029** | .009 | 3.386 | [.012, .047] | .001 | **.005** | .003 | 1.972 | [.000, .010] | .049 |
| | High | **.024** | .012 | 2.057 | [.001, .047] | .040 | .001 | .004 | .252 | [-.006, .008] | .801 |
| Medium | Low | .012 | .008 | 1.489 | [-.004, .027] | .136 | **.011** | .002 | 4.640 | [.007, .016] | < .001 |
| | Medium | .006 | .007 | .854 | [-.008, .020] | .393 | **.007** | .002 | 3.757 | [.003, .011] | < .001 |
| | High | .000 | .009 | .040 | [-.018, .018] | .968 | .003 | .003 | .982 | [-.003, .009] | .326 |
| High | Low | -.012 | .014 | -.842 | [-.040, .016] | .400 | **.013** | .003 | 4.466 | [.007, .019] | < .001 |
| | Medium | -.018 | .013 | -1.349 | [-.043, .008] | .177 | **.009** | .002 | 4.018 | [.005, .014] | < .001 |
| | High | -.023 | .014 | -1.705 | [-.050, .003] | .088 | .005 | .003 | 1.638 | [-.001, .011] | .101 |

*Note*. *Est.* = unstandardized estimate; significant estimates are shown in bold. MM = mind-mindedness; PosControl = positive control. Regression slopes were computed at low (1 *SD* below the *mean*), medium (*mean*), and high (1 *SD* above the *mean*) levels of each moderator within site. ENG = England; MC = mainland China.

**Figure S1.** *Residuals vs. fitted plots testing linearity of associations between parental distress and mind-mindedness*

A. England ($n$ = 253) B. Hong Kong ($n$ = 260) C. Mainland China ($n$ = 324)

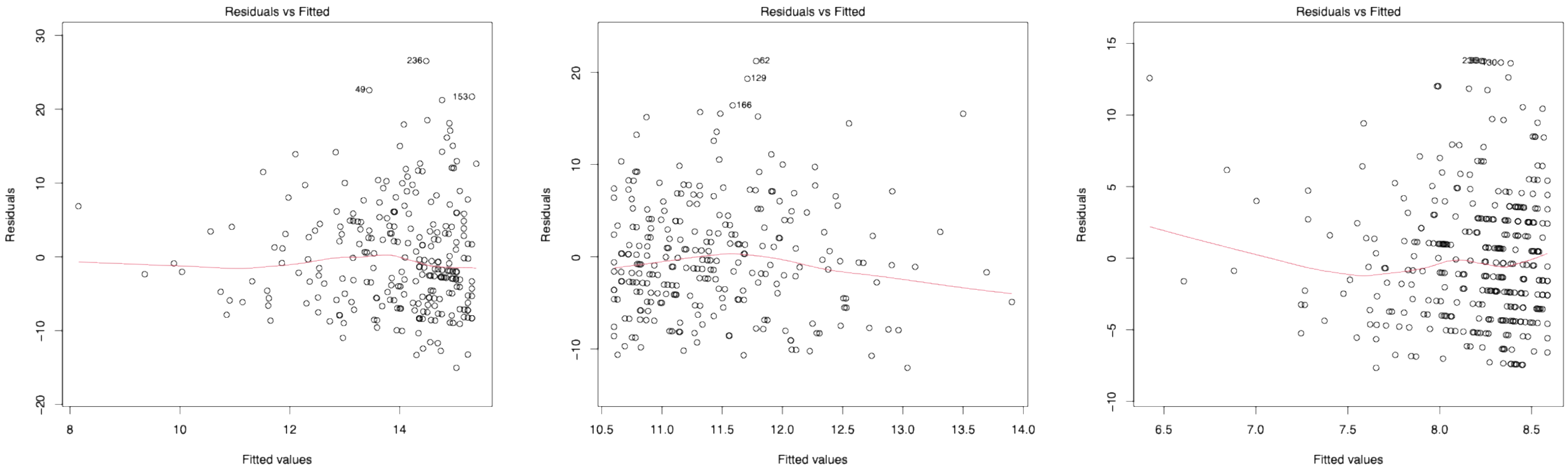


*Note*. Labeled data points represent observations with relatively large standardized residuals. The random distribution of residuals around zero suggests that the assumption of linearity is reasonably met across all sites.

**Figure S2.** *Residuals vs. fitted plots testing linearity of associations between parental distress and positive control*

A. England (*n* = 221) B. Hong Kong (*n* = 237) C. Mainland China (*n* = 324)

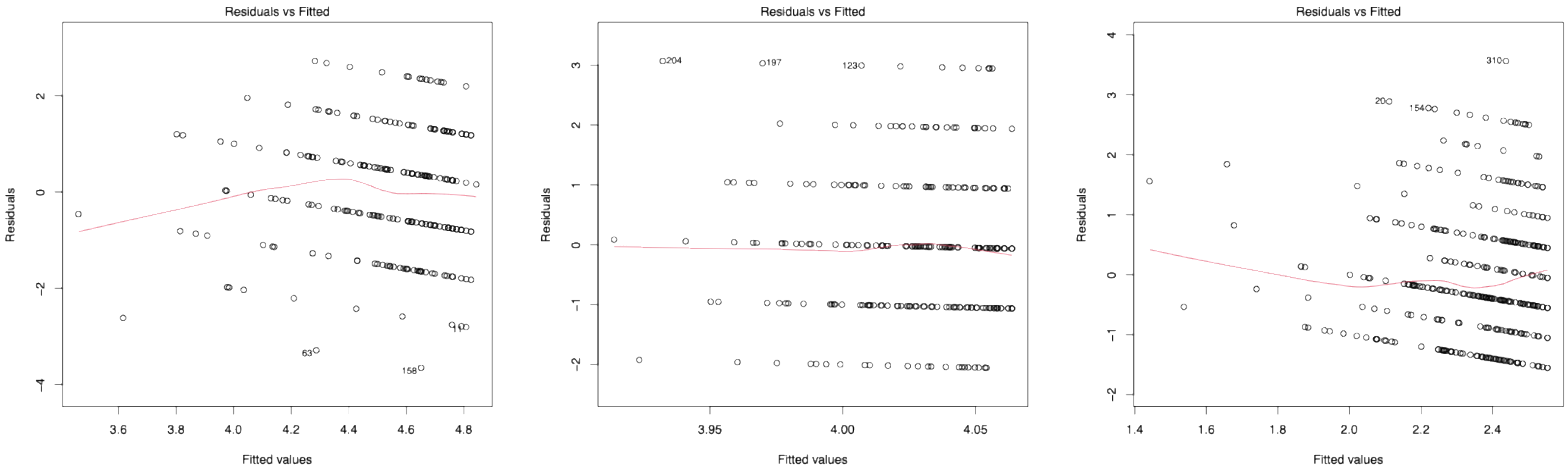


*Note*. Labeled data points represent observations with relatively large standardized residuals. The random distribution of residuals around zero suggests that the assumption of linearity is reasonably met across all sites.

**Figure S3.** *Forest plot displaying consistent effect sizes for the link between parental distress and positive control across sites*

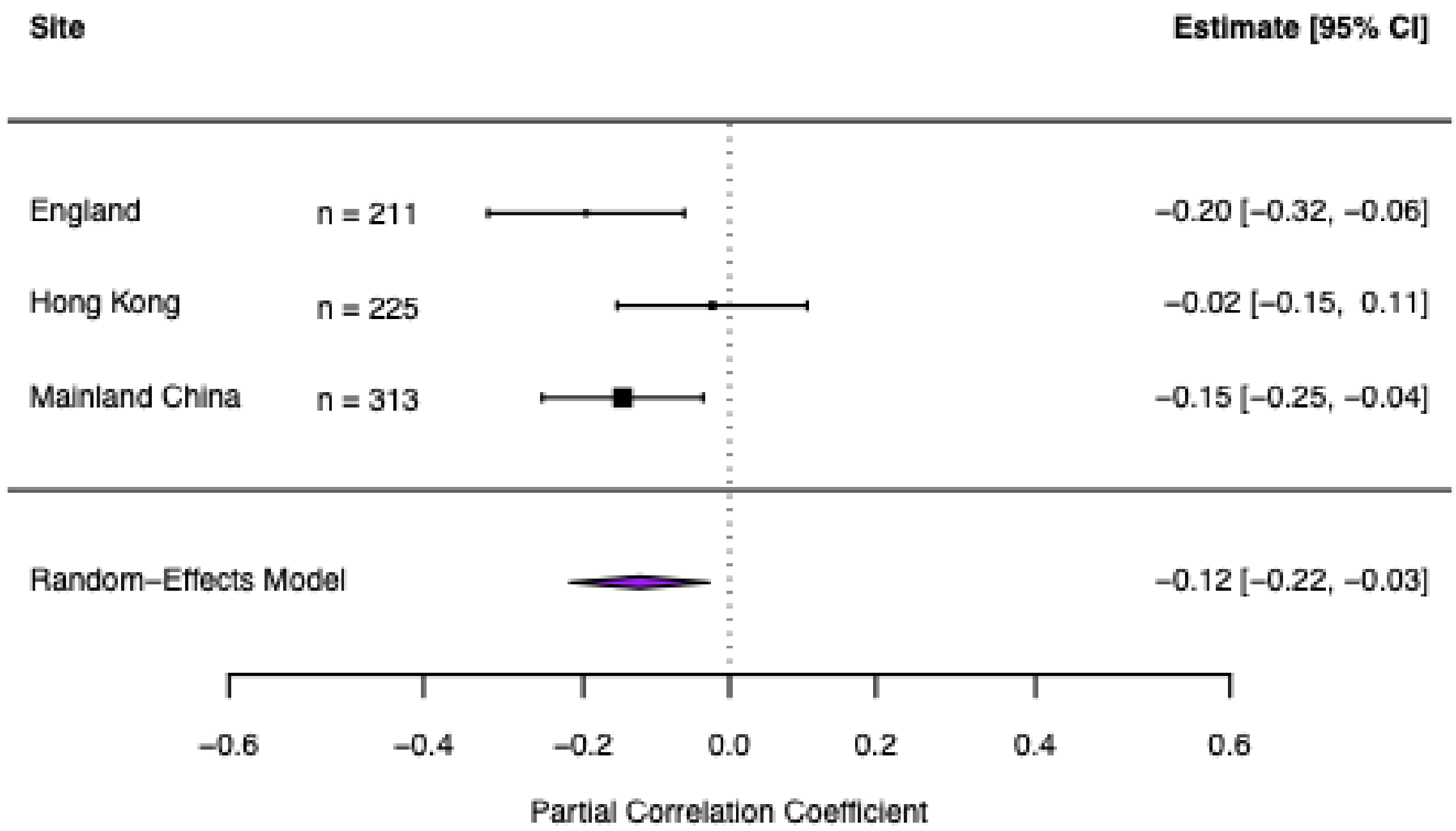


*Note*. Bars represent 95% confidence intervals; square size reflects relative sample size; the diamond represents the overall effect estimated using a random-effects model.

**Figure S4.** *Forest plot displaying varying effect sizes for the link between parental distress and mind-mindedness across sites*

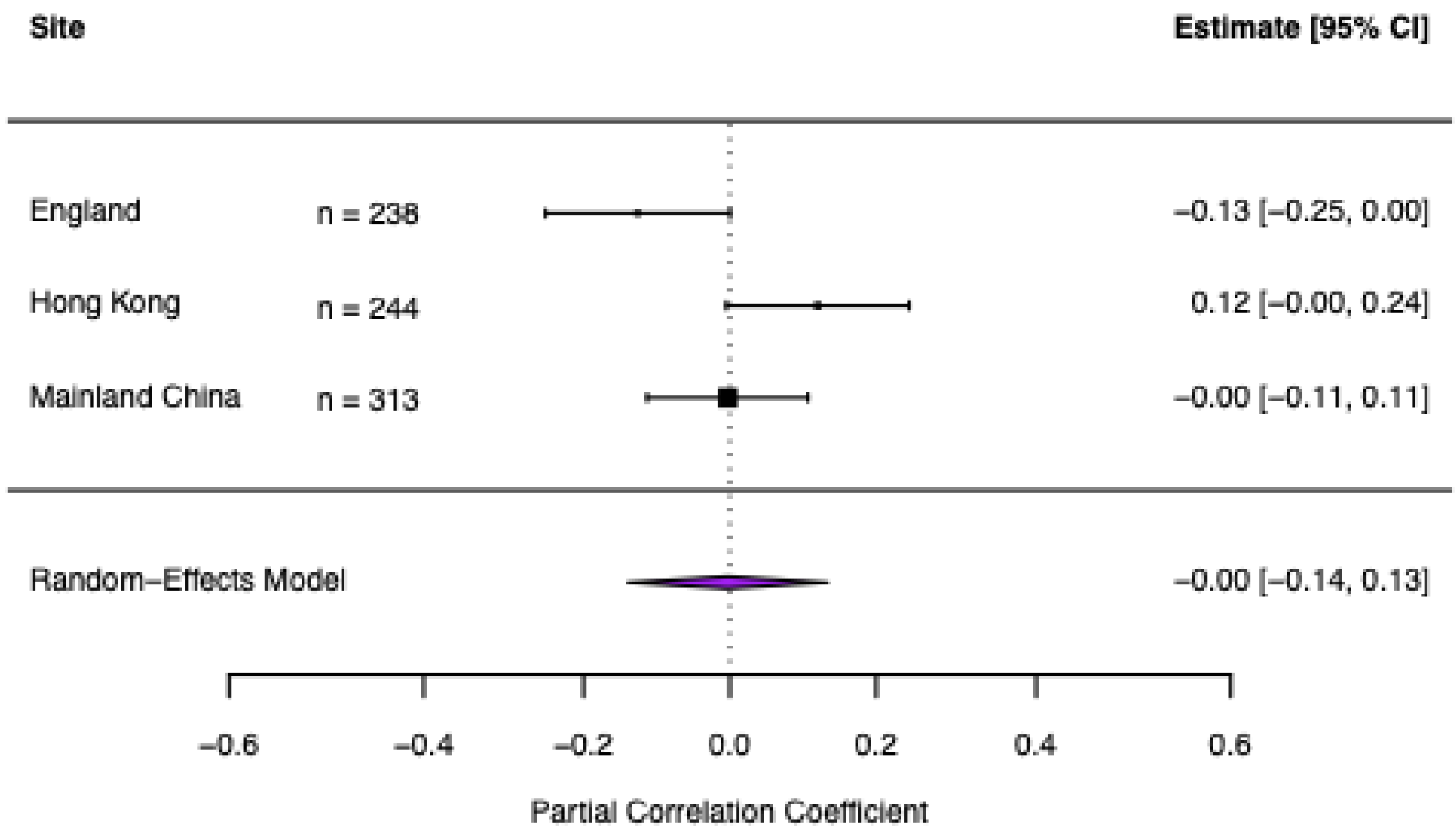


*Note*. Bars represent 95% confidence intervals; square size reflects relative sample size; the diamond represents the overall effect estimated using a random-effects model.
